# Metformin Restores CNS Remyelination Capacity by Rejuvenating Aged Stem Cells

**DOI:** 10.1016/j.stem.2019.08.015

**Published:** 2019-10-03

**Authors:** Björn Neumann, Roey Baror, Chao Zhao, Michael Segel, Sabine Dietmann, Khalil S. Rawji, Sarah Foerster, Crystal R. McClain, Kevin Chalut, Peter van Wijngaarden, Robin J.M. Franklin

**Affiliations:** 1 Wellcome – MRC Cambridge Stem Cell Institute, Jeffrey Cheah Biomedical Centre, Cambridge Biomedical Campus, University of Cambridge, Cambridge, CB2 0AW, UK; 2 Centre for Eye Research Australia, Royal Victorian Eye and Ear Hospital, Melbourne, Australia; 3 Ophthalmology, Department of Surgery, University of Melbourne, Melbourne, Australia; 4 Department of Physics, University of Cambridge, Cambridge CB3 0HE, UK

**Keywords:** aging, remyelination, CNS regeneration, adult stem cell, oligodendrocyte progenitor cell, rejuvenation, metformin, dietary restriction

## Abstract

The age-related failure to produce oligodendrocytes from oligodendrocyte progenitor cells (OPCs) is associated with irreversible neurodegeneration in multiple sclerosis (MS). Consequently, regenerative approaches have significant potential for treating chronic demyelinating diseases. Here, we show that the differentiation potential of adult rodent OPCs decreases with age. Aged OPCs become unresponsive to pro-differentiation signals, suggesting intrinsic constraints on therapeutic approaches aimed at enhancing OPC differentiation. This decline in functional capacity is associated with hallmarks of cellular aging, including decreased metabolic function and increased DNA damage. Fasting or treatment with metformin can reverse these changes and restore the regenerative capacity of aged OPCs, improving remyelination in aged animals following focal demyelination. Aged OPCs treated with metformin regain responsiveness to pro-differentiation signals, suggesting synergistic effects of rejuvenation and pro-differentiation therapies. These findings provide insight into aging-associated remyelination failure and suggest therapeutic interventions for reversing such declines in chronic disease.

## Highlights

• Aged OPCs fail to respond to differentiation signals
• Aged OPCs acquire many of the hallmarks of cell aging
• Fasting and the fasting mimetic metformin rejuvenate poor remyelination in aged rodents
• Metformin reverses age-related changes, making OPCs respond to differentiation factors

## Graphical Abstract

**Figure:**
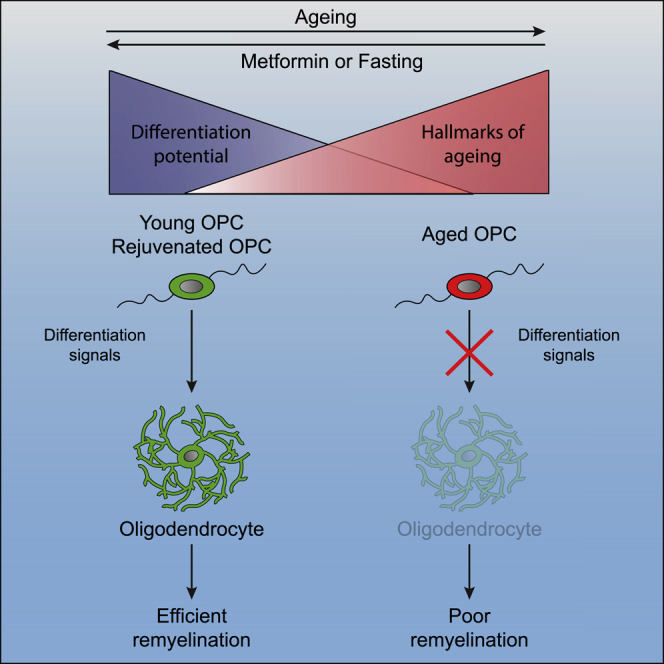

## Teaser

Neumann et al. demonstrate that aging renders adult oligodendrocyte progenitor cells unresponsive to pro-differentiation factors, which can be reversed both *in vitro* and *in vivo* by calorie restriction (CR) and the CR mimetic metformin.

## Introduction

The ability to regenerate oligodendrocytes, the myelin-forming cells of the CNS, contrasts with the poor capacity to regenerate neurons in most brain regions (Franklin and Ffrench-Constant, 2017). Generation of oligodendrocytes from oligodendrocyte progenitor cells (OPCs) occurs throughout life and contributes to myelin turnover (Young et al., 2013, Hill et al., 2018, Hughes et al., 2018, Tripathi et al., 2017) and adaptive myelination (Gibson et al., 2014, Mitew et al., 2018, Hughes et al., 2018) as well as to the regenerative process of remyelination that follows demyelination (Zawadzka et al., 2010). As with most regenerative processes, the efficiency of remyelination declines progressively with aging to the extent that it becomes so slow that it eventually fails (Oh et al., 2014, Sim et al., 2002). This has important implications for chronic demyelinating diseases such as multiple sclerosis (MS) that can extend over several decades. Delayed remyelination renders demyelinated axons susceptible to irreversible degeneration, a phenomenon that underpins the progressive neurological decline associated with the later stages of MS (Franklin et al., 2012).

The slowing of remyelination with aging is characterized by impaired recruitment of OPCs into the lesion area as well as their delayed differentiation into oligodendrocytes (Sim et al., 2002). Chronically demyelinated MS lesions either lack OPCs or, more commonly, contain OPCs that have failed to differentiate (Boyd et al., 2013, Chang et al., 2002, Kuhlmann et al., 2008). Increasing the number of OPCs in white matter lesions of aged animals does not improve remyelination (Woodruff et al., 2004), indicating that differentiation of OPCs into oligodendrocytes is the bottleneck for remyelination. The mechanisms that regulate OPC differentiation are dysregulated in the aging brain (Shen et al., 2008), in part because of age-related changes in the cells and molecules in the environment in which remyelination occurs (Cantuti-Castelvetri et al., 2018, Hinks and Franklin, 2000, Natrajan et al., 2015). These changes can be overcome, in principle, by providing a more youthful systemic environment that is permissive for regeneration (Ruckh et al., 2012). Thus, remyelination can potentially be enhanced by pro-differentiation factors lacking in the aged brain. However, it remains unclear whether OPCs undergo intrinsic changes with aging that affect their responsiveness to differentiation signals.

## Results

### Aged OPCs Differentiate Slowly and Do Not React to Pro-differentiation Compounds

We first asked whether age-related changes in OPCs contribute to the differentiation delay observed in aged animals during remyelination (Sim et al., 2002). Studies of OPC aging have been hampered by the technical challenges of culturing OPCs isolated from the aged adult rodent CNS. Thus, we first optimized existing protocols to establish cultures of adult OPCs from young adult (2–3 months) and aged (20–24 months) rats (Figure S1; Dugas and Emery, 2013). We used magnetic activated cell sorting (MACS) for A2B5 to isolate cells from young and aged adult brains (Figure S1A). The positively selected cells showed minor contamination for CD11b^+^ microglia (<0.8%) or MOG^+^ oligodendrocytes (<2%) with no significant difference between preparations from young and aged animals (Figures S1B–S1D). Using immunohistochemistry, we found that A2B5^+^ cells of both age groups co-expressed PDGFRa, NG2, Sox10, and Olig2, confirming their identity as OPCs (Figures S1E–S1I and S1N–S1R). In contrast, A2B5^+^ cells from young or aged adults never expressed mature lineage markers such as CNPase and MBP (Figures S1J and S1K), the microglia marker Cd11b (Figure S1L), or the astrocyte marker GFAP (Figure S1M), indicating that cultures were free of mature oligodendrocytes, microglia, and astrocytes. This enabled us to compare the differentiation efficiency of OPCs isolated from the young adult and aged CNS.

**Figure 1:**
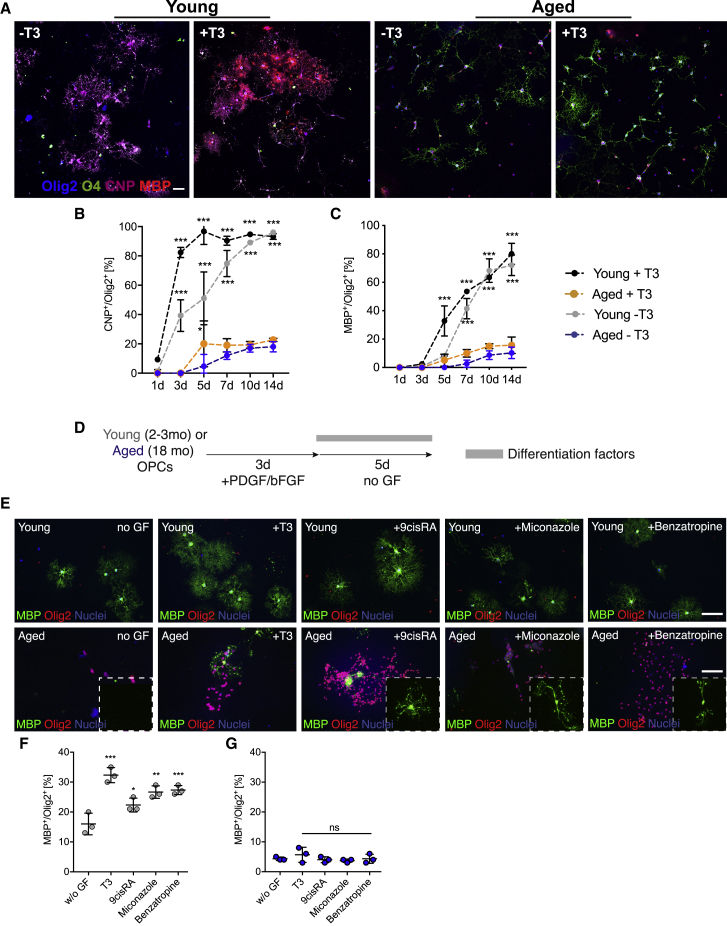
OPCs Lose Their Inherent Capacity for Differentiation and Their Responsiveness to Differentiation Factors with Aging (A) Representative images of young adult (2–3 months old) and aged OPCs (20–24 months old) differentiated in the absence of growth factor or in the presence of T3. Increasing maturity was visualized using O4 (early), CNPase (intermediate), and MBP (mature), immunocytochemical markers of the oligodendrocyte (OL) lineage. Scale bars, 50 μm. (B and C) Quantification of cells over time in culture: CNPase^+^/Olig2^+^ cells (B) and MBP^+^/Olig2^+^ cells (C). Statistical significance was determined using two-way ANOVA repeated measurements followed by Dunnett’s post test to compare each group against “aged T3.” All data are presented as mean ± SD (n = 3 biological repeats). (D) Schematic of the experimental design. (E) Representative images of the differentiation assay performed with young and aged OPCs. Newly formed oligodendrocytes were identified as MBP^+^/Olig2^+^ cells. Scale bars, 50 μm. (F and G) Quantification of the differentiation assay for young (F) and aged OPCs (G). n = 3 biological replicates for each group, one-way ANOVA with Dunnett’s multiple comparisons test for each group against the group differentiating in the absence of growth factors (“w/o GF”). Error bars represent SDs. ^∗^p < 0.05, ^∗∗^p < 0.01, ^∗∗∗^p < 0.001. See also Figures S1–S3.

We next tested the relative differentiation ability of OPCs from young adults (hereafter referred to as young OPCs) and OPCs from old adults (aged OPCs) when grown in differentiation medium from which the growth factors that maintain proliferation were removed. Although 60% of young OPCs differentiated into mature oligodendrocytes (CNPase^+^ and MBP^+^), fewer than 20% of aged OPCs acquired these markers within the same period, revealing a slower inherent capacity for differentiation (Figures 1A–1C). We next assessed how adult OPCs responded to thyroid hormone (T3), a well-established promoter of OPC differentiation (Gao et al., 1998). Although T3 accelerated the differentiation of young OPCs, there was no significant effect on the differentiation of aged OPCs (Figures 1B and 1C). Similar results were obtained with other factors known to have pro-differentiation effects on OPCs derived from newborn animals or on pluripotent stem cells, such as 9-*cis*-retinoic acid (Huang et al., 2011), miconazole (Najm et al., 2015), and benzatropine (Deshmukh et al., 2013), all of which enhanced differentiation in young adult (Figures 1E and 1F) but not aged OPCs (Figures 1E, 1G, and S4).

Cultures of aged OPCs were generally of lower cell density, suggesting decreased survival. Because the density might influence the differentiation rate through secretion of paracrine factors, we asked whether increasing the cellular density might rescue the differentiation ability of aged OPCs or reduce the differentiation rate of young OPCs. Seeding half of the normal number of young OPCs or twice the number of aged OPCs did not result in a significant change in differentiation rates (Figures S2A–S2E), indicating that the differences in cellular density between young and aged cultures do not account for the reduced differentiation of aged OPCs.

Because some aged OPCs differentiated into MBP^+^ oligodendrocytes, we asked whether the non-differentiating cells were unable to differentiate or were differentiating at a slower rate. To test this, we cultured aged OPCs for 4 weeks in differentiation medium and found a significant increase in differentiation (Figures S2D and S2E), indicating that aged OPCs do not lose their general ability for differentiation but undergo intrinsic changes that significantly slow their differentiation program. These intrinsic changes also cause aging OPCs to become less responsive to factors that induce differentiation, which likely contributes to the failure of oligodendrocyte lineage differentiation, characteristic of many non-remyelinating chronic MS lesions (Kuhlmann et al., 2008).

### Aged OPCs Exhibit Classical Hallmarks of Aging

**Figure 2:**
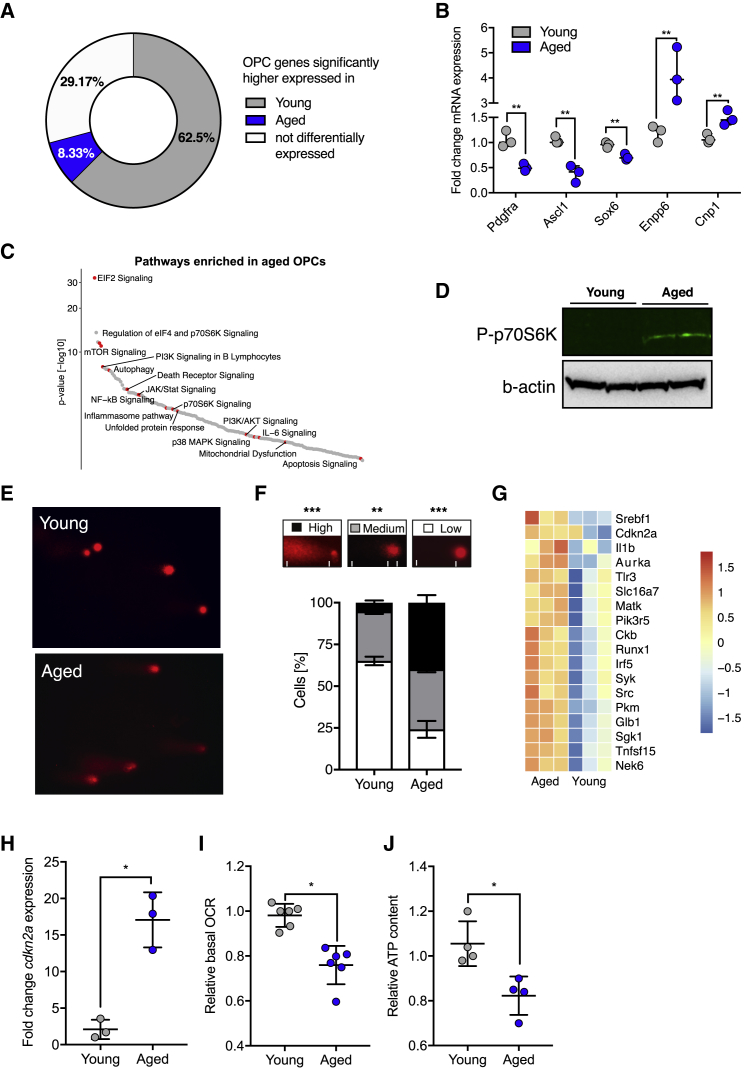
Aged OPCs Have Reduced Expression of OPC-Specific Genes and Acquire Hallmarks of Aging (A) Young and aged OPCs were tested for differential expression of OPC-specific genes. The pie chart summarizes the findings as the percentage of genes that were expressed at significantly higher levels in aged or young OPCs (p.adj < 0.05) or that were not differentially expressed (p.adj > 0.05). See also Table S1. (B) qRT-PCR validation of several genes identified in RNA-seq, comparing freshly isolated young and aged OPCs (n = 3 biological replicates for each age group, two-tailed t test). (C) Top 5 pathways identified by ingenuity pathway analysis (*Z* score > 2 and p.adj. < 0.05) for genes enriched in aged OPCs (p.adj < 0.05; see also Table S2). (D) Western blot for the downstream mTORC1 pathway target p70S6K and actin loading controls. P, phosphorylated; n = 2 biological samples for each age group. (E) Representative images for comet assays (alkaline conditions) of freshly isolated young and aged OPCs to visualize the degree of DNA damage. Presence of a tail indicates DNA damage. (F) Quantification of the comet assay. The categories used for scoring are depicted in the respective boxes. Statistical significance was determined using one-way ANOVA and Turkey’s post test. All data are presented as mean ± SD (n = 3 biological replicates for each age group). (G) Heatmap of genes from RNA-seq data whose expression is associated with cellular senescence. All depicted genes are differentially expressed (n = 3 biological repeats). (H) qRT-PCR results visualizing expression of the senescence marker *Cdkn2a*. Data are presented as mean ± SD (n = 3 biological replicates for each age group, two-tailed t test). (I) Fold change of the basal oxygen consumption rate (bOCR), measured after overnight culture *in vitro* (n = 3 biological repeats for each age group, two-tailed t test). (J) Normalized intracellular ATP content of freshly isolated OPCs (n = 5 biological repeats for each age group, two-tailed t test). Error bars represent SDs. ^∗^p < 0.05, ^∗∗^p < 0.01, ^∗∗∗^p < 0.001.

Next we characterized molecular alterations responsible for the aged OPC phenotype using RNA sequencing (RNA-seq) to compare the transcriptomes of OPCs isolated from young and aged rats. Approximately 20% of all genes were differentially expressed with aging (1.5-fold change in expression, adjusted p value [p.adj] < 0.05). Among the genes more highly expressed in young adult OPCs were those that are characteristic of adult OPCs in 21-day-old mice (Marques et al., 2016; Figure 2A) and their self-renewal, including *Pdgfra*, *Ascl1*, and *Ptprz1* (Emery, 2010; Figure 2B; .Table S1). In contrast, aged OPCs expressed higher levels of the early differentiation markers *Cnp1*, *Sirt2*, and *Enpp6* (Figure 2B). Because we did not find a higher proportion of MOG^+^ cells or those expressing more mature lineage markers, such as CNPase, in our aged OPC preparations compared with young OPCs (Figures S1J and S1K), we ruled out the possibility that these changes in the transcriptome were caused by contamination with oligodendrocytes. Thus, we concluded that aged OPCs lose their characteristic stem cell signature (Figures 2A and 2B). To identify the cellular processes that might contribute to the aged OPC state, we used ingenuity pathway analysis on genes preferentially expressed in aged OPCs. We found enrichment of terms that are closely linked to organismal and stem cell aging, such as mitochondrial dysfunction, unfolded protein response (UPR), autophagy, inflammasome signaling, and nuclear factor κB (NF-κB and p38 mitogen-activated protein kinase (MAPK) signaling (Figure 2C). Consistent with the predictions made on the basis of the RNA-seq data, we found increased mTOR activity in freshly isolated aged OPCs by detection of the phosphorylated forms of the downstream target p70S6-kinase (Figure 2D). mTOR activity is a crucial regulator of adult stem cell quiescence, activation, and differentiation (Mihaylova et al., 2014, Rodgers et al., 2014) and is linked to cellular aging (Laplante and Sabatini, 2012). Aging is associated with increased and dysregulated mTOR activity, which contributes to DNA damage and cellular senescence (Castilho et al., 2009, Chen et al., 2009, Yilmaz et al., 2006). We therefore predicted that both DNA damage and markers of senescence would increase with adult OPC aging. Consistent with this prediction, single-cell comet assays revealed that aged OPCs had significantly more DNA damage than young OPCs (Figures 2E and 2F). Using our RNA-seq data, we also found that aged OPCs expressed several genes associated with cellular senescence at significantly higher levels than young OPCs (Figure 2G; Tacutu et al., 2018). We found that aged OPCs had 8-fold higher mRNA levels of the senescence marker *Cdkn2a* (Figure 2H). Last, aged OPCs had lower levels of ATP and reduced cellular respiration (Figures 2I and 2J), likely reflecting a combination of mitochondrial dysfunction and reduced mitochondrial content. Thus, aged OPCs, like other adult stem cells, acquire a variety of hallmarks of aging that likely contribute to loss of their regenerative potential.

### Alternate-Day Fasting Enhances Remyelination in Aged Rats through Functional Rejuvenation of OPCs

**Figure 3:**
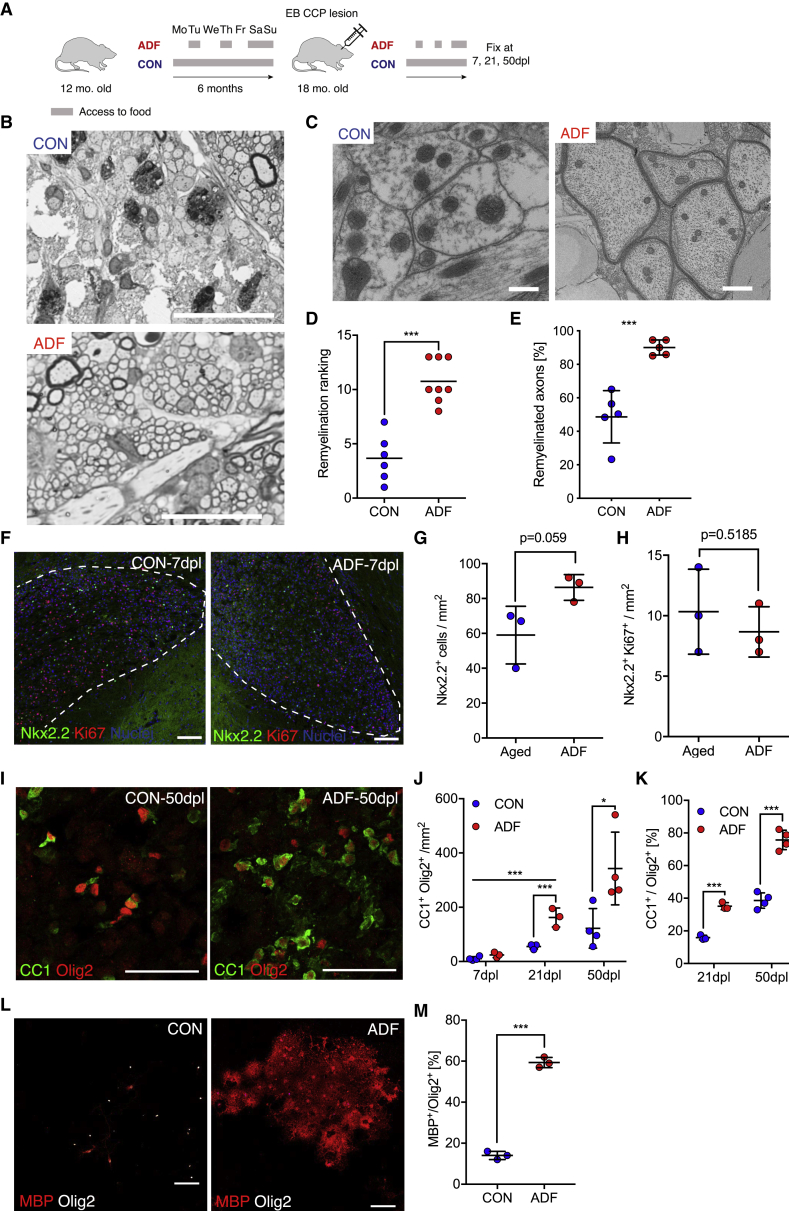
ADF Enhances Remyelination in Aged Rats Partially through Restoration of the Differentiation Capacity of OPCs (A) Schematic of the fasting experiment. ADF animals had access to food on alternate days only. Control animals had free access to food (*ad libitum*). Fasting was initiated at 12 months of age for 6 months. White matter demyelination was induced by focal injection of ethidium bromide (EtBr) into the caudal cerebellar peduncle (CCP). (B) Remyelination was assessed 50 days post-lesion induction in semi-thin resin sections stained with toluidine blue. Remyelination is evident as dark circles surrounding a pale gray axon. Myelinated axons that have not undergone demyelination are surrounded by thick, dark myelin. Demyelinated axons have poorly discernible borders. Scale bars, 100 μm. (C) Electron micrographs from areas within the lesion center. Scale bars, 0.5 μm. (D) Quantification of the remyelination data. Each dot represents a single animal. The rank corresponds to the extent of remyelination; a higher rank indicates better remyelination (n ≥ 6 for each group, Mann-Whitney *U* test). (E) Percentage of remyelinated axons (n ≥ 4 for each group). (F) OPCs were identified as Nkx2.2^+^ cells within the lesion area (dashed line). Proliferating cells are labeled with Ki67. Scale bars, 100 μm. (G) Quantification of the number of Nkx2.2^+^ OPCs within the lesion at 7 days post lesion (dpl). (H) Quantification of the density Ki67^+^ OPCs at 7 dpl (n = 4 biological replicates). (I) Representative images of differentiating oligodendrocytes (Olig2^+^/CC1^+^ cells) within the lesion center at 50 dpl. (J) Quantification of the density of newly formed oligodendrocytes within the lesion at 7 dpl, 21 dpl. and 50 dpl. (K) Quantification of the proportion of newly formed oligodendrocytes among all lineage cells (Olig2^+^). (L) Differentiation assay of OPCs that were isolated from 18-month-old animals that had undergone dietary restriction or had free access to food as described in (A). Differentiated oligodendrocytes were visualized on day 10 of differentiation as MBP^+^/Olig2^+^ cells. Scale bars, 50 μm. (M) The proportion of all oligodendrocyte lineage cells (Olig2^+^) that are differentiated oligodendrocytes MBP+/Olig2^+^. n = 3 for ADF 21 dpl and n = 4 biological replicates for all other groups in (G), (H), (J), and (K), two-tailed t test; n = 3 biological replicates for each group for (I), two-tailed t test. Error bars represent SDs. ^∗^p < 0.05; ^∗∗^p < 0.01; ^∗∗∗^p < 0.001; ns, p > 0.05. See also Figures S4 and S5.

On the basis of these findings, we hypothesized that reversal of these age-related changes in OPCs might be necessary to reinstate their differentiation potential. We therefore explored strategies known to alter the effects of aging as a potential strategy to improve OPC function and remyelination in aged animals. Dietary restriction is the most effective intervention known to alter the organismal aging process (Fontana and Partridge, 2015) and it does so in part by enhancing the function of aged adult stem cells (Cerletti et al., 2012, Mihaylova et al., 2018). To test the effect of dietary restriction on remyelination, we subjected 12-month-old rats, an age when remyelination rate is substantially slower than in young adult rats (Sim et al., 2002, Shields et al., 1999), to alternate-day fasting (ADF) for 6 months (Figure 3A). We confirmed that OPCs from 12-month-old rats, like those from 24-month-old rats, were impaired in their differentiation and unable to respond to differentiation signals (Figure S3). At the end of the 6-month fasting period, we induced demyelination in cerebellar white matter by focal injection of ethidium bromide, a well-established *in vivo* model for studying remyelination (Woodruff and Franklin, 1999). We assessed the degree of remyelination 50 days post lesion (dpl) induction in semi-thin resin sections stained with toluidine blue, in which remyelinated axons are easily identifiable by their thin myelin sheaths (Blakemore, 1974; Figure 3B), which was confirmed by examination of electron micrographs (Figure 3C). Although remyelination in *ad libitum*-fed animals was restricted to the border of the lesion, animals undergoing fasting consistently exhibited nearly complete remyelination (Figures 3B and 3C). The clear difference in the extent of remyelination between the two groups was reflected by a blind ranking analysis of semi-thin sections of lesions as well as quantification of the percentage of remyelinated axons within the lesions (Figures 3B, 3D, and 3E). We also compared g ratios of control and treatment groups but found no differences, suggesting that the effect of ADF is on the extent rather than the quality of remyelination (Figure S5A). To further explore fasting-enhanced remyelination in aged animals, we characterized the recruitment, proliferation, and differentiation of OPCs during remyelination. At 7 dpl, there was no difference in the density of OPCs within lesions of animals subjected to fasting or *ad libitum* feeding (Figures 3F–3H). However, the density of mature oligodendrocytes (Olig2^+^/CC1^+^) was 2-fold greater in the lesions of fasting animals compared with those of controls at both 21 and 50 dpl (Figures 3I and 3J). The proportion of Olig2^+^ cells expressing CC1 was significantly higher in lesions of fasting rats at both time points relative to controls, suggesting enhanced OPC differentiation (Figure 3K).

The positive effects of fasting regimens on adult stem cells has been attributed to alterations within the stem cells and to changes in the stem cell niche or in the systemic environment (Cerletti et al., 2012, Igarashi and Guarente, 2016, Lee et al., 2000, Mihaylova et al., 2018, Yilmaz et al., 2012). We therefore reasoned that the observed effect of fasting on remyelination is likely the consequence of changes in a variety of cell types (such as astrocytes, inflammatory cells, and vascular cells as well as OPCs) associated with the lesion, caused by changes in the systemic environment. To determine whether the enhanced differentiation capacity of aged OPCs in fasting animals was at least in part due to intrinsic alterations within the OPCs, we isolated OPCs from unlesioned ADF and control aged animals. We found that OPCs derived from fasting animals differentiated into MBP^+^ oligodendrocytes more rapidly than those from age-matched control rats (Figures 3L and 3M) and with efficiency comparable with OPCs from young rats (Figure 1). Moreover, OPCs derived from fasting animals expressed higher levels of OPC self-renewal genes (Figure S4A), lower levels of differentiation genes (Figure S4A), and lower levels of *Cdkn2a* (Figure S4B); exhibited less DNA damage (Figures S4C and S4D); showed reduced levels of phosphorylated p70S6K (Figure S4E); and had higher ATP levels (Figure S4F). Thus, we concluded that ADF restored remyelination in part through the functional rejuvenation of aged OPCs. To address whether effects on other cell types also contributed to enhanced remyelination in ADF animals, we assessed the density of GFAP^+^ astrocytes and Iba1^+^ innate immune cells within the lesion during remyelination. In neither case was there a significant change between samples from fasting and control animals (Figures S5B–S5E). However, we found that removal of myelin debris was improved in animals undergoing fasting (Figures S5F–S5H), suggest functional changes in phagocytic cells within the lesion and a potential mechanism contributing to the improved remyelination outcome in ADF animals (Ruckh et al., 2012).

### The Fasting Mimetic Metformin Restores the Differentiation Capacity of Aged OPCs

Because dietary restriction protocols are difficult to translate into a clinical context, we asked whether the small-molecule fasting mimetic metformin might replicate the effects of fasting. The fasting-mimicking effects of metformin, a drug widely prescribed for type 2 diabetes, are mediated via modulation of the AMPK pathway, a central nutrient signaling pathway. We found that metformin-treated cells exhibited increased expression of *Pdgfra* and *Ascl1* (Figures S4G and S4H), expressed significantly less *Cdkn2a* (Figure S4I), and had less DNA damage, as indicated by comet assays (Figure S4J), suggesting that metformin is sufficient to ameliorate some of the hallmarks of aging and phenocopy at least some of the effects of ADF (Figures S4A–S4F).

**Figure 4:**
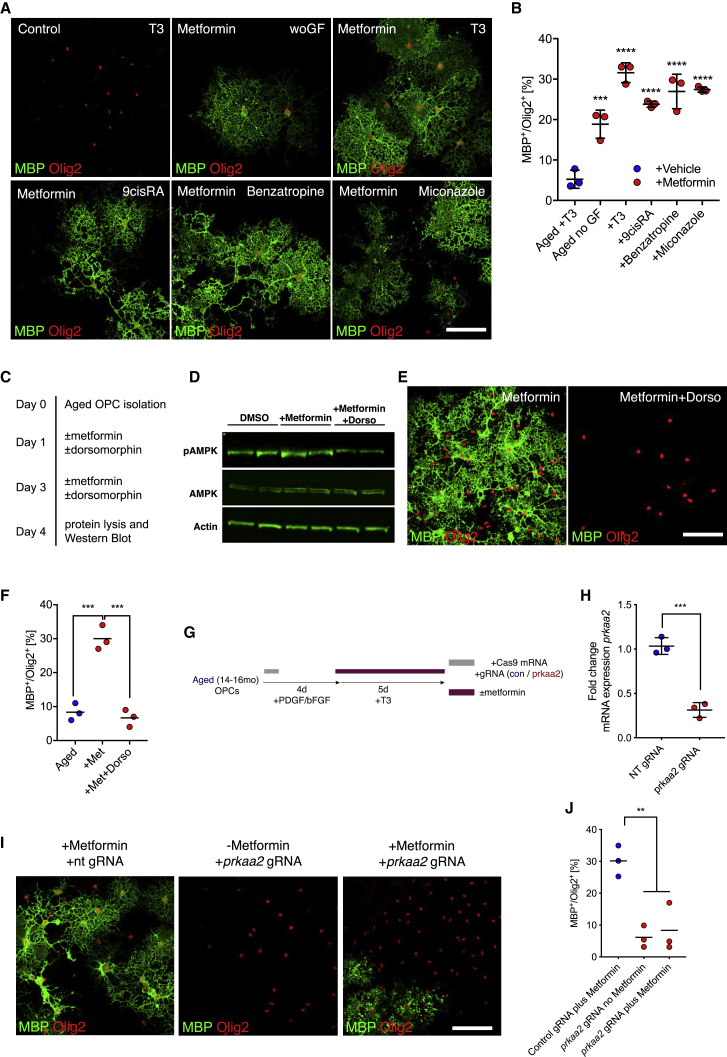
Metformin Restores the Ability of Aged OPCs to Differentiate in an AMPK-Dependent Manner (A) Representative images of differentiation assays. Newly formed oligodendrocytes are identified as MBP^+^/Olig2^+^ cells. Scale bar, 50 μm for all images. Prior to differentiation, OPCs were isolated from aged animals (≥18 months) and cultured in the presence of growth factors for 5 days. Some cells were treated with 100 μM metformin with each medium change during the first 5 days (days 2 and 4). (B) Quantification of the differentiation assay as the proportion of MBP^+^ cells among all lineage cells (Olig2^+^). All data are presented as mean ± SD (n = 3 biological replicates, one-way ANOVA with Dunnett’s multiple comparisons test against aged OPCs culture in the absence of growth factors [aged no GF]). (C) Schematic illustration. (D) Western blots for AMPK pathway proteins from aged OPCs treated with DMSO, 100 μM metformin, or 100 μM metformin and 1 μM dorsomorphin (labeled Dorso) for 3 days *in vitro*. P, phosphorylated (n = 2 biological repeats for each group.) (E) Representative images of differentiation assay findings. Aged OPCs were treated with metformin alone or with metformin and dorsomorphin during the first 5 days of culture, similar as described in Figure S5G. Newly formed oligodendrocytes are identified as MBP^+^/Olig2^+^ cells. Scale bars, 50 μm. (F) Proportion of Olig2^+^ in the differentiation assay that are differentiated (MBP^+^/Olig2^+^). All data are presented as mean ± SD (n = 3 biological replicates, one-way ANOVA with Dunnett’s multiple comparisons test against “+met”). (G) Schematic for experiments to knock out *Prkaa2* (AMPK) in aged OPCs using CRISPR/Cas9. (H) qPCR confirming knockout of prkaa2 mRNA after CRISPR with guide RNAs (gRNAs) targeting *Prkaa2* (n = 3 biological repeats). (I) Representative images of differentiation assays after CRISPR-mediated knockout of *Prkaa2*. Newly formed oligodendrocytes are identified as MBP^+^/Olig2^+^ cells. Scale bars, 50 μm. (J) Quantification of the differentiation assay in (H) (n = 3 biological repeats, all data are mean ± SD, two-tailed t test). met, metformin; dorso, dorsomorphin. Error bars represent SDs. ^∗^p < 0.05; ^∗∗^p < 0.01; ^∗∗∗^p < 0.001; ns, p > 0.05. See also Figures S4 and S6.

**Figure 5:**
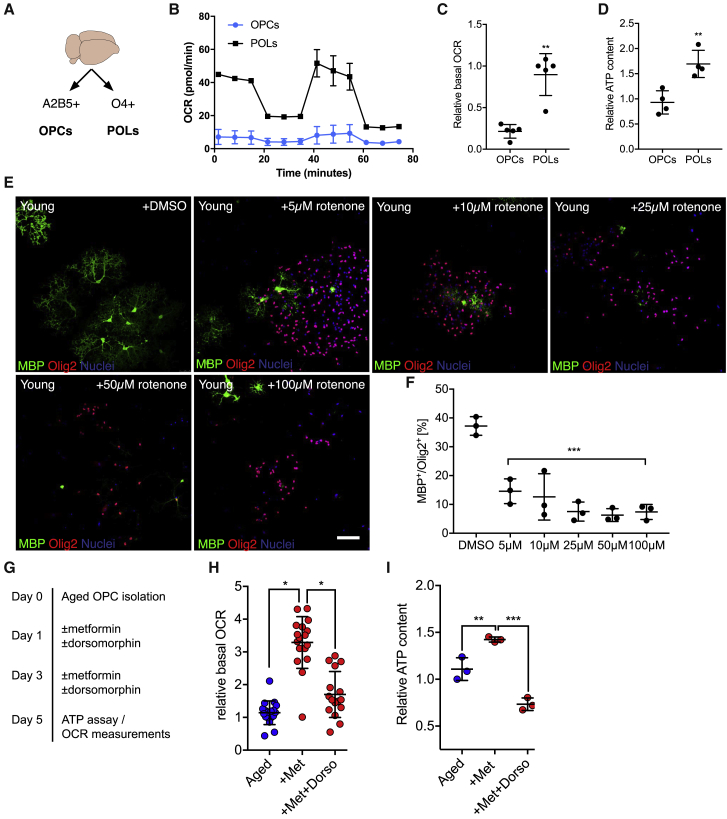
Mitochondrial ATP Production Is Required for OPC Differentiation (A) OPCs and differentiating pre-oligodendrocytes (POLs) were isolated from young rats (2–3 months) by MACS using A2B5 (OPCs) and O4 (pre-OL) antibodies. (B) Representative graph depicting the fold change of the bOCR of OPCs and POLs under basal conditions and sequential treatment with oligomycin, carbonyl cyanide-4-(trifluoromethoxy)phenylhydrazone (FCCP), and rotenone and antimycin A. (C) Quantification of the bOCR normalized to POL (n = 2 biological replicates for each group, two-tailed t test). (D) Quantification of normalized ATP measurements from freshly isolated OPCs and POLs. (n = 2 biological repeats for each group, circles depict technical repeats, two-tailed t test). (E) Representative images of differentiation cultures from young OPCs after treatment with DMSO or increasing concentrations of rotenone (a mitochondrial complex I blocker). Newly formed oligodendrocytes were identified as MBP^+^/Olig2^+^ cells. Scale bar, 50 μm. (F) Quantification of the differentiation assay (all data are presented as mean ± SD; statistical significance was determined using one-way ANOVA with Dunnett’s post test for each treatment group against DMSO; n = 3 biological replicates). (G) Schematic illustration of the experiments using metformin and dorsomorphin. (H) Relative change in the bOCR, measured after treatment of aged OPCs with metformin and/or dorsomorphin for 5 days *in vitro*. Dots represent technical replicates (n = 3 biological repeats for each age group, two-tailed t test). (I) Intracellular ATP content of aged OPCs, normalized to cell numbers in the respective control group, treated with metformin alone or metformin and dorsomorphin (n = 3 biological repeats for each age group, two-tailed t test). Error bars represent SDs. ^∗^p < 0.05; ^∗∗^p < 0.01; ^∗∗∗^p < 0.001; ns, p > 0.05.

We then asked whether exposure to metformin would also be sufficient to restore the potential of aged OPCs to differentiate and restore the responsiveness of aged OPCs to differentiation factors. Treatment with metformin prior to onset of differentiation increased the number of differentiated cells and enhanced the responsiveness of aged OPCs to pro-differentiation factors (Figures 4A and 4B), indicating functional rejuvenation of aged OPCs, but had no effect on differentiation of young OPCs (Figure S6). The mechanisms by which metformin rejuvenates aging OPCs are likely to be multiple but, at least in part, appear to be through the AMPK pathway because metformin increases the levels of phosphorylated AMPK (Figures 4C and 4D), whereas inhibition of AMPK signaling by dorsomorphin, a small-molecule inhibitor of the AMPK pathway, abrogated the effect of metformin on AMPK phosphorylation and aged OPC differentiation (Figures 4C, 4E, and 4F). Because dorsomorphin is also known to affect other kinases, we additionally used a genetic approach to specifically disrupt signaling through AMPK. Using the CRISPR/Cas9 system, we found that aged OPCs transfected with guide RNAs targeting *Prkaa2* (AMPK) did not show an increased rate of differentiation in response to treatment with metformin (Figures 4G–4J), suggesting that restoration of the differentiation capacity was dependent on AMPK. A potential downstream mechanism could be modulation of mitochondrial function. We found that young OPCs had a lower basal oxygen consumption rate as well as lower intracellular ATP levels compared with O4^+^ pre-oligodendrocytes (Figures 5B–5D), suggesting that OPC differentiation is accompanied by an increase in mitochondrial function. Treatment of young OPCs with rotenone, a mitochondrial complex I inhibitor, impaired differentiation, indicating that mitochondrial function is required for this process (Figures 5E and 5F). Because mitochondrial function was reduced in aged cells compared with young cells (Figures 2I and 2J), and because AMPK signaling influences mitochondrial function, we hypothesized that metformin treatment might enhance mitochondrial function in aged OPCs. We found that metformin treatment increased cellular respiration and ATP levels in aged OPCs (Figures 5G–5I), effects that were lost with addition of dorsomorphin. These data suggested that improved mitochondrial function in aged OPCs following metformin treatment contributed to the restored differentiation capacity.

### Metformin Enhances Remyelination in Aged Rats

**Figure 6:**
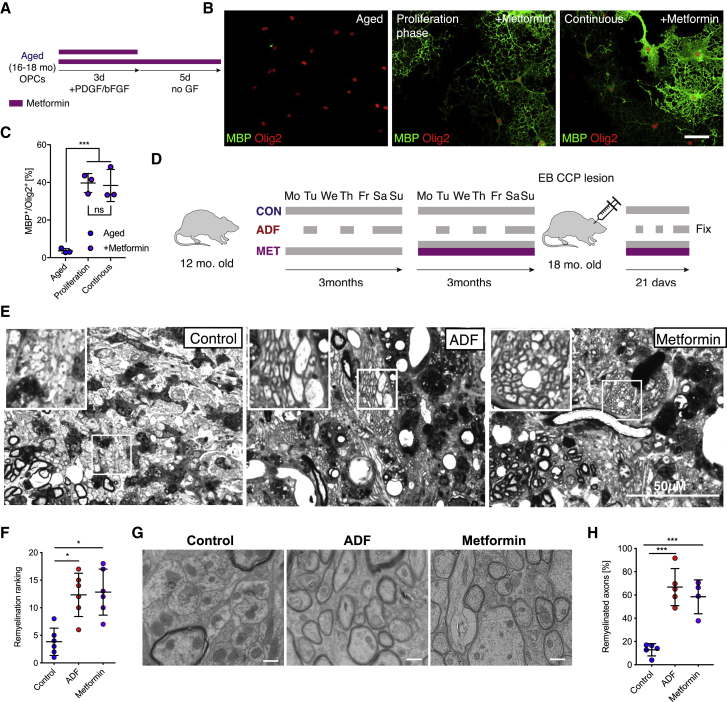
Metformin Treatment Enhances Remyelination in Aged Rats (A) Aged OPCs were treated with metformin either during the recovery period prior to differentiation or during the entire culture period. (B) Representative images of differentiation cultures at 5 days of differentiation. Newly formed oligodendrocytes were identified as MBP^+^/Olig2^+^ cells. Scale bar, 50 μm. (C) Quantification of the number of differentiated Olig2^+^ MBP^+^ cells after 5 days of differentiation (all data are presented as mean ± SD; statistical significance was determined using one-way ANOVA with Dunnett’s test for multiple comparisons against group “Aged”; n = 3 biological replicates). (D) 12-month-old-female SD rats were divided into three groups. The control and ADF groups were treated as described in Figure 3A. Metformin animals had *ad libitum* access to food but received metformin at dose of 300 mg/kg bodyweight in their drinking water from the age of 15 months. At 18 months of age, demyelinating lesions were induced by injection of EtBr into the CCP. (E) Remyelination is evident as dark circles surrounding a pale gray axon. Myelinated axons that have not undergone demyelination are surrounded by thick, dark myelin. Demyelinated axons have poorly discernible borders. Scale bar, 50 μm. (F) Ranking analysis of remyelination. Each dot represents a single animal. The rank corresponds to the degree of remyelination; a higher rank indicates better remyelination (n = 6 biological replicates for each group, Kruskal-Wallis test followed by Dunn’s post test). (G) Representative electron micrographs of lesions. Scale bars, 500 nm. (H) Quantification of the percentage of remyelinated axons (n ≥ 4 biological repeats for each group, one-way ANOVA with multiple comparisons between each group). Error bars represent SDs. ^∗^p < 0.05; ^∗∗^p < 0.01; ^∗∗∗^p < 0.001; ns, p > 0.05.

Lesions in MS occur in an unpredictable manner and are not synchronized. This also implies that the regenerative process will not be synchronized within an individual. Therefore, any treatment targeting OPCs must not interfere with any specific stage of the remyelination process. We therefore asked whether the timing of metformin exposure during OPC differentiation might influence its effect. We found that exposure to metformin throughout the entire culture period produced the same effect as pre-incubation of aged OPCs with the drug before onset of differentiation by removal of platelet-derived growth factor (PDGF) and basic fibroblast growth factor (bFGF) (Figures 6A–6C). Finally, we tested whether metformin treatment could mimic ADF *in vivo*. Aged rats fed *ad libitum* received metformin in their drinking water for 3 months prior to induction of a focal demyelinating injury and during the time after lesion induction (Figure 6D). Metformin-treated animals exhibited remyelination that was comparable in extent with animals undergoing ADF and significantly higher than that in control animals, as judged by the ranking analysis and percentage of remyelinated axons (Figures 6E–6H), although there was no difference in g ratios of remyelinated axons between the groups (Figure S4).

## Discussion

MS is a chronic degenerative disease that typically becomes progressive. Epidemiological data indicate that age more than the duration of the disease influences when MS becomes progressive (Confavreux and Vukusic, 2006). The progressive phase is characterized by accumulative neurodegeneration. This is most likely the result of an unmet need for repair and a concomitant increase in the susceptibility of neurons and their axons for degeneration, which are both processes closely tied to aging. Thus, understanding how aging affects remyelination and how these effects may be reversed is critical to devising regenerative interventions with which to combat progressive MS.

Studies of OPC differentiation pathways have identified a number of candidate therapeutic agents to enhance remyelination (Deshmukh et al., 2013, Hubler et al., 2018, Mei et al., 2014, Najm et al., 2015), but none have been tested in aged cells or aged animals. This is important because aging is associated with remyelination failure, and age-related changes in stem cell function may render cells less responsive to candidate therapies. Indeed, our findings reveal that OPCs do indeed lose the capacity to respond to endogenous differentiation cues and candidate therapeutic agents with increasing age, with significant implications for therapies that aim to enhance remyelination.

Difficulties encountered in isolating and culturing OPCs from aged animals have previously limited our understanding of the influence of aging on these cells. Here we report methods for successful isolation and culture of OPCs from aged rats. Transcriptional analyses and *in vitro* assays revealed that aged OPCs exhibit many hallmarks of stem cell aging, including DNA damage and reduced mitochondrial function, which are known to impede cellular function (Oh et al., 2014). Importantly, using fasting or metformin to ameliorate hallmarks of aging, we show that the age-dependent loss of OPC potential is reversible, which is a pre-requisite for pharmacological interventions targeting endogenous OPCs to stimulate remyelination. Because exposure of metformin to purified cultures of aged OPCs led to functional rejuvenation, it is plausible that metformin could also have exerted a direct effect on OPCs *in vivo*. We show a direct effect of metformin on OPCs in culture and present evidence that this effect is AMPK-dependent and associated with an increase in mitochondrial function (Figure 4), which is required for differentiation (Figure 5). However, this is unlikely to be the only mechanism orchestrating this complex process because metformin has multiple functions, such as stimulating DNA repair pathways and activating autophagy. Even enhancement of a single cellular processes alone can lead to amelioration of several hallmarks of aging. For example, overexpression of Atg7, a key component of the autophagy pathway, improves proteostasis, as expected, but also improves mitochondrial function and reduces the levels of senescence markers (García-Prat et al., 2016). Similarly, the reduction of reactive oxygen species (ROS) in aged muscle stem cells by supplementation of an antioxidant has the same effects on these hallmarks of aging (Ho et al., 2017). The question that follows is whether restoring the function of aged OPCs alone could be sufficient to enhance remyelination in an aged animal.

Experiments in other stem cell systems indicate that rejuvenation of aged adult stem cells by inhibition of p38-MAPK signaling and exposure to a softer culture surface can be sufficient to restore regenerative ability in an aged animal (Cosgrove et al., 2014). Thus, it is possible that rejuvenation of aged OPCs by metformin is sufficient to promote remyelination in aged animals following demyelinating injuries. In addition, treatments such as metformin and fasting have wide-ranging systemic effects that together may be permissive for remyelination. For example, metformin is known to reduce inflammasome signaling, and reductions in this signaling have been reported to enhance remyelination in middle-aged mice (Cantuti-Castelvetri et al., 2018). Further, fasting has been shown to enhance muscle stem cell function and muscle regeneration relative to *ad libitum* feeding, even in young animals (Cerletti et al., 2012). It follows that the enhanced remyelination observed with metformin treatment is likely a composite of direct effects on OPCs together with effects on other cell types. Our *in vitro* work indicates that metformin acts synergistically with drugs identified previously to promote differentiation of OPCs. Importantly, when used in the absence of metformin treatment, these candidate remyelination drugs failed to mediate differentiation of aged OPCs. This points to the need for combination therapies to enhance remyelination *in vivo* because remyelination failure typically occurs in the context of aging.

Overcoming the effects of aging on OPCs is important in generating a permissive environment for remyelination. Interventions such as dietary restriction or drugs that mimic its effects will likely alter the function of OPCs and other cell types that contribute to remyelination. In addition, fasting and calorie restriction mimetics have been shown to suppress autoimmunity-mediated demyelination (Choi et al., 2016, Cignarella et al., 2018, Piccio et al., 2008) and may thus have dual benefits for the treatment of chronic demyelinating neurodegenerative diseases.

## STAR★Methods

### Key Resources Table

**Table:** 

REAGENT or RESOURCE	SOURCE	IDENTIFIER
**Antibodies**		

Anti-Olig2	Millipore	Cat# AB15328; RRID:AB_2299035
Anti-Sox-10 (N-20)	Santa Cruz Biotechnologies	Cat# sc-17342; RRID:AB_2195374
Anti-CD140a (Pdgfra)	BD Biosciences	Cat# 558774; RRID:AB_397117
Anti-NG2 Chondroitin Sulfate Proteoglycan	Millipore	Cat# AB5320; RRID:AB_11213678
Anti-A2B5, clone A2B5-105	Millipore	Cat# MAB312; RRID:AB_94709
Anti-Ki 67	Abcam	Cat# ab16667; RRID:AB_302459
Anti-APC (Ab-7) Mouse mAb (CC-1)	Millipore	Cat# OP80; RRID:AB_2057371
Anti-Oligodendrocyte Marker O4	R&D Systems	Cat# MAB1326; RRID:AB_357617
Anti-CNPase [11-5B]	Abcam	Cat# ab6319; RRID:AB_2082593
Anti-MBP (aa82-87)	Bio-rad	MCA409S; RRID:AB_325004
Anti-Nkx2.2	DSHB	Cat# 74.5A5; RRID:AB_531794
Anti-Rat-Cd11b	Bio-rad	Cat# MCA275R; RRID:AB_321302
Anti-GFAP	Abcam	Cat# ab53554; RRID:AB_880202
Anti-A2B5-PE	Milteny Biotec	Cat# 130-098-038; RRID:AB_2660799
Anti-Mouse IgM-PE	Milteny Biotec	Cat# 130-099-127; RRID:AB_2661766
Anti MOG Biotinylated	R&D Systems	Cat# BAF2439; RRID:AB_2145536
Mouse anti-rat Cd11b	Novus Bioscience	Cat# NB100-2143; RRID:AB_10001618
Anti-rat CD11b/c PerCP/Cy5.5	Biolegend	Cat# 201819; RRID:AB_2565948
PerCP/Cy5.5 Mouse IgG2a, kappa Isotype Ctrl	Biolegend	Cat# 400257; RRID:AB_10695169
SAv-Brilliant Violet 421	Biolegend	Cat# 405226
Donkey anti-Mouse IgG (H+L) Highly Cross-Adsorbed Secondary Antibody, Alexa Fluor 488	Thermo Fisher Scientific	Cat# A-21202; RRID:AB_141607
Donkey anti-Mouse IgG (H+L) Highly Cross-Adsorbed Secondary Antibody, Alexa Fluor 594	Thermo Fisher Scientific	Cat# A-21203; RRID:AB_2535789
Goat anti-Mouse IgM Heavy Chain Cross-Adsorbed Secondary Antibody, Alexa Fluor 488	Thermo Fisher Scientific	Cat# A-21042; RRID:AB_2535711
Donkey anti-Mouse IgG (H+L) Highly Cross-Adsorbed Secondary Antibody, Alexa Fluor 647	Thermo Fisher Scientific	A-31571; RRID:AB_162542
Donkey anti-Rabbit IgG (H+L) Highly Cross-Adsorbed Secondary Antibody, Alexa Fluor 488	Thermo Fisher Scientific	Cat# A-21206; RRID:AB_2535792
Donkey anti-Rabbit IgG (H+L) Highly Cross-Adsorbed Secondary Antibody, Alexa Fluor 594	Thermo Fisher Scientific	Cat# A-21207; RRID:AB_141637
Donkey anti-Goat IgG (H+L) Cross-Adsorbed Secondary Antibody, Alexa Fluor 647	Thermo Fisher Scientific	Cat# A-21447; RRID:AB_2535864
Donkey anti-Rat IgG (H+L) Highly Cross-Adsorbed Secondary Antibody, Alexa Fluor 488	Thermo Fisher Scientific	Cat# A-21208; RRID:AB_2535794
Donkey anti-Rat IgG (H+L) Highly Cross-Adsorbed Secondary Antibody, Alexa Fluor 594	Thermo Fisher Scientific	Cat# A-21209; RRID:AB_2535795
Donkey anti-Goat IgG (H+L) Cross-Adsorbed Secondary Antibody, Alexa Fluor 488	Thermo Fisher Scientific	Cat# A-11055; RRID:AB_2534102
Goat anti-Mouse IgG2b Cross-Adsorbed Secondary Antibody, Alexa Fluor 555	Thermo Fisher Scientific	Cat# A-21147; RRID:AB_2535783
Anti-AMPK alpha 1 + AMPK alpha 2	Abcam	Cat# ab80039; RRID:AB_1603618
Anti-Phospho-AMPKα (Thr172) (40H9)	Cell Signaling Technology	Cat# 2535
Anti-p70 S6 Kinase	Cell Signaling Technology	Cat# 9202; RRID:AB_331676
Anti-Phospho-p70 S6 Kinase (Thr389)	Cell Signaling Technology	Cat# 9205; RRID:AB_330944
Anti-S6 Ribosomal Protein (54D2)	Cell Signaling Technology	Cat# 2317; RRID:AB_2238583
Anti-Phospho-S6 Ribosomal Protein (Ser235/236) (D57.2.2E)	Cell Signaling Technology	Cat# 4858; RRID:AB_916156
Anti-beta-Actin Peroxidase conjugated	Sigma-Aldrich	Cat# A3854; RRID:AB_262011
Donkey Anti-Mouse IgG Antibody, IRDye® 680LT	LI-COR Biosciences	Cat# 926-68022; RRID:AB_10715072
Donkey Anti-Rabbit IgG Antibody, IRDye® 680LT	LI-COR Biosciences	Cat# 926-68023; RRID:AB_10706167
Donkey Anti-Mouse IgG, IRDye 800CW	LI-COR Biosciences	Cat# 926-32212; RRID:AB_621847

**Chemicals, Peptides, and Recombinant Proteins**		

Recombinant Human FGF-basic	Peprotech	100-18B
Recombinant Human PDGF-AA	Peprotech	100-13A
Insulin, human recombinant, zinc solution	Thermo Fisher Scientific	Cat# 12585014
Sodium pyruvate	Thermo Fisher Scientific	*11360070*
apo-Transferrin human	Sigma	Cat# T1147
Putrescine	Sigma	p5780
Sodium Selenite	Sigma	S-5261
Progesterone	Sigma	P8783
Bovine Serum Albumin	Sigma	A4919
T3	Sigma	T6397
Dorsomorphin	LC Laboratories	D-3197
Metformin	Sigma	PHR1084-500MG
Benztropine mesylate	Sigma	SML0847
9-cis-retinoic acid	Sigma	R4643
Miconazole	Sigma	M3512
Isolation medium / (alternatively Hibernate A w/o Mg and Ca)	This study / Brainbits	Table S3 / HACAMG500
Antimycin A	Sigma	A8674-25MG
FCCP	Sigma	C2920-10MG
Oligomycin	Sigma	495455-10MG
Red blood cell lysis buffer	Sigma	Cat# R7757
Percoll	GE Healthcare	17-0891-01
Lipofectamine LTX	Thermo Fisher Scientific	15338030

**Critical Commercial Assays**		

ATPlite luminescence assay system	Perkin Elmer	6016943
Seahorse FluxPak XFp	Agilent	103022-100
Seahorse FluxPak XFe96	Agilent	102601-100

**Deposited Data**		

RNaseq data of young and aged OPCs	This study	GEO: GSE134765

**Experimental Models: Cell Lines**		

Adult oligodendrocyte progenitor cells	This study	STAR Methods text

**Experimental Models: Organisms/Strains**		

Sprague Dawley rats	Charles River and in house breeding	RRID:MGI:5651135

**Oligonucleotides**		

*Prkaa2* gRNA1 Forward primer for gRNA generation: AAGCTAATACGACTCACTATAGACCAT ACGACATTATGGCGGGTTTTAGAGCTAGA	This study	This study
*Prkaa2* gRNA2 Forward primer for gRNA generation: AAGCTAATACGACTCACTATAGTGCTCATCG TCGAACGGGAGTTTTAGAGCTAGA	This study	This study
Non targeting gRNA Forward primer for gRNA generation: AAGCTAATAC GACTCACTATAGAAATGTGAGATCA GATAATTTTAGAGCTAGA	This study	This study
Scaffold for gRNA Reverse primer for gRNA generation: AAAAGCACCGACTCGG TGCCACTTTTTCAAGTTGATAACG GACTAGCCTTATTTTAACTTGCTAT TTCTAGCTCTAAAAC	This study	This study
Cas9 Forward primer to generate template for *in vitro* transcription: TAATAC GACTCACTATAGGCCACCATGTAC CCATACGA	This study	This study
Cas9 Reverse primer to generate template for *in vitro* transcription: GAATTCTTAGCTGGCCTCCAC	This study	This study
qPCR primers	Sigma	Table S5

**Recombinant DNA**		

AAV-CMVc-Cas9	Addgene (deposited by Juan Belmonte’s laboratory)	plasmid # 106431

**Software and Algorithms**		

TrimGalore	https://github.com/FelixKrueger/TrimGalore	RRID:SCR_016946
TopHat2	http://ccb.jhu.edu/software/tophat	RRID:SCR_013035
feature counts (Subread)	http://subread.sourceforge.net	RRID:SCR_009803
DESeq2 (Version 1.16.1)	https://bioconductor.org/packages/release/bioc/html/DESeq2.html	RRID:SCR_015687
Ingenuity Pathway analysis	https://www.ingenuity.com/products/pathways_analysis.html	RRID:SCR_008653
Pheatmap (Version 1.0.8)	https://github.com/raivokolde/	RRID:SCR_016418
R (Version 3.4.4)	http://www.r-project.org	RRID:SCR_001905
FIJI	http://fiji.sc	RRID:SCR_002285
Graph Pad Prism 8	https://www.graphpad.com/	RRID:SCR_002798
FlowJo	https://www.flowjo.com/solutions/flowjo	RRID:SCR_008520

### Lead Contact and Materials Availability

Further information and requests for resources and reagents should be directed to and will be fulfilled by the Lead Contact, Robin JM Franklin (rjf1000@cam.ac.uk). This study did not generate new unique reagents.

### Experimental Model and Subject Details

#### Animal husbandry

All animal procedures were performed in compliance with United Kingdom Home Office regulations. The animals were housed under standard laboratory conditions on a 12 h light/dark cycle with constant access to food and water. All animals were housed in pairs or groups of up to 4 animals.

For alternate day fasting (ADF) 12 months old female SD rats were restricted from food every other day. ADF animals had access to food on Tuesday, Thursday, Saturday and Sunday and all food was removed from their cages on Monday, Wednesday and Friday. The food was removed and returned in the mornings. The weight of each animal was weekly monitored. The fasting paradigm was interrupted for the first three days after surgery when all animals had free access to food.

For metformin treatment 15 months old female SD rats that were fed *ad libitum* received metformin (Glucophage) in their drinking water (300mg/kg bodyweight per day). Metformin treatment was interrupted for two days before and three days after surgery and then commenced to the end of the study (21 days after lesion induction). Fluid consumption was continuously monitored to adapt dosages. All animals were randomly allocated to experimental groups.

#### Isolation of adult oligodendrocyte progenitor cells

Adult male and female rats (2-24 months) were decapitated after lethal injection with phenobarbital. The brains were removed quickly and placed into ice-cold isolation medium (Table S3; alternatively Hibernate A Brainbits). The telencephalon and cerebellum were dissected in isolation medium; meninges, and the olfactory bulb were mechanically removed and the brain tissue was mechanically minced into 1mm^3^ pieces. The tissue pieces were spun down at 100 g for 1min at RT and the tissue was washed in HBSS- (no Mg2+ and Ca^2+^, GIBCO). Each half of the brain was mixed with 5ml of dissociation solution (34U/ml papain (Worthington), 20 μg/ml DNase Type IV (GIBCO) in isolation medium). The brain tissue was dissociated on a shaker (50rpm) for 40 min at 35°C. The digestion was stopped by addition of ice cold HBSS-. The tissue was centrifuged (200 g, 3 min, RT), the supernatant completely aspirated and the tissue resuspended in isolation medium supplemented with 2% B27 and 2mM sodium-pyruvate (trituration solution). The tissue was allowed to sit in this solution for 5min. To obtain a single cell suspension the tissue suspension was triturated 10 times using first a 5ml serological pipette and subsequently three fire polished glass pipettes (opening diameter > 0.5mm). After each trituration step the tissue suspension was allowed to sediment (approximately 1-2 min) and the supernatant (approximately 2ml), containing the cells, was transferred into a fresh tube. After each round of trituration 2ml of fresh trituration solution were added. To remove accidentally transferred undigested tissue bits, the collected supernatant was filtered through 70 μm cell strainers into tubes that contained 90% isotonic Percoll (GE Healthcare, 17-0891-01, in 10xPBS pH7.2 (Lifetech). The final volume was topped up with phenol-red free DMEM/F12 with HEPES (GIBCO) and mixed to yield a homogeneous suspension with a final Percoll concentration of 22.5%. The single cell suspension was separated from remaining debris particles by gradient density centrifugation (800 g, 20min, RT, without break). The myelin debris and all layers without cells were discarded and the brain cell containing phase (last 2ml) and cell pellet were resuspended in HBSS+ and combined in a fresh 15ml tubes and centrifuged (300 g, 5min, RT). The cell pellet was resuspended in red blood cell lysis buffer (Sigma, R7757) and incubated for 1min at RT to remove red blood cells. 10ml of HBSS+ were added to this cell suspension and spun down (300 g, 5min, RT). The cell pellets were resuspended in 0.5ml modified Milteny washing buffer (MWB, 2mM EDTA, 2mM Na-Pyruvate, 0.5% BSA in PBS, pH 7.3) supplemented with 10ng/ml human recombinant insulin (GIBCO). To this cell suspension 2.5μg mouse-anti-rat-A2B5-IgM antibody (Millipore, Table S4) were added for every 10 million cells. After 25 min incubation, gently shaking at 4°C, 7ml of MWB were added. The solution was centrifuged (300 g, 5min, RT) and the pellet resuspended in 80 μL MWB supplemented with 20 μL rat-anti-mouse-IgM antibody (Milteny, 130-047-302) per 10 million cells. The cells were incubated for 15 min, slowly shaking at 4°C. The secondary antibody was again washed out with 7ml MWB and the sample was centrifuged (300 g, 5min, RT). The cell pellet was resuspended in 0.5ml and MACS was performed according to the recommendations of the supplier. Briefly, a MS column (Milteny, 130-042-201) were inserted into MiniMACS Separator (Miltenyi; 130-042-102) and pre-wet with 0.5ml MWB. Resuspended cells were put onto one MS column. Subsequently the column was washed three times using 500 μL MWB for each wash. Finally, A2B5 positive cells were flushed out the column with 1ml pre-warmed, CO_2_ and O_2_ pre-equilibrated OPC medium.

#### Culture of adult oligodendrocyte progenitor cells

Isolated OPCs were seeded onto 12mm glass coverslips in 24 well plates (VWR) or into 96 well-plates (InVitro-Sciences) coated with PDL (Sigma). After isolation, OPCs were left to recover in OPC medium (60μg/ml N-Acetyl cysteine (Sigma), 10μg/ml human recombinant insulin (GIBCO), 1mM sodium pyruvate (GIBCO), 50μg/ml apo-transferrin (Sigma), 16.1μg/ml putrescine (Sigma), 40ng/ml sodium selenite (Sigma), 60ng/ml progesterone (Sigma), 330μg/ml bovine serum albumin (Sigma)) supplemented with b-FGF and PDGF (30ng/ml each, Peprotech). OPCs were incubated at 37°C, 5% CO_2_ and 5% O_2_. The medium was completely exchanged to OPC medium with 20ng/ml bFGF and PDGF after overnight culture to remove any dead cells. After 3d the cell culture medium was switched to promote further proliferation (OPC medium+20ng/ml bFGF and PDGF) or differentiation (OPCM + 40ng/ml T3). During differentiation or proliferation experiments 66% of the medium were replaced every 48h and growth factors or other small molecules were added fresh to the culture. The culture medium used was 500 μL for cultures in 24 well plate wells and 150 μL for cultures in 96 well plate wells. For differentiation assays the medium was in some instances supplemented with 40ng/ml thyroid-hormone (T3, Sigma), 50nM 9-*cis* retinoic acid (9cRA, Sigma), 1μM miconazole (Sigma, M3512) or 1.5μM benztropine (Sigma, SML0847). Otherwise used small molecules: 100μM metformin (Sigma), rotenone (Sigma, R8875), 1μM dorsomorphin (LC Laboratories, D-3197).

### Method Details

#### Induction of white matter lesions and assessment of remyelination

For studies involving demyelination, female Sprague Dawley rats (Harlan Laboratories) 18 months of age were used. The rats were anesthetized with buprenorphine (0.03mg/kg, s.c.) and 2.5% isoflurane. Demyelination was induced by stereotaxic injection of 4μl of 0.01% ethidium bromide (EB) into the caudal cerebellar peduncles (CCPs), as previously described (Woodruff and Franklin, 1999). EB was delivered at a rate of 1 μl/min. After EB delivery, the injection needle remained in position for additional 4 min.

To assess remyelination the rats were transcardially perfused with 4% glutaraldehyde and 0.4 mM CaCl_2_ in PBS. The cerebellum was cut in to transverse 1mm thick sections. The tissue was fixed in 2% osmium-tetroxide at 4°C overnight, dehydrated through a series of washes in ethanol and propylene-oxide and embedded in resin. From the resin blocks 1μm thick sections were cut and stained with 1% toluidine blue. To compare the extent of remyelination in different experimental groups we first used a blinded ranking analysis. Single blocks from each animal from which sections containing the largest area of lesion were identified and used for subsequent analysis. In resin sections, remyelinated axons can be readily distinguished from normally myelinated axons outside the lesion by the thinness of the myelin sheath. Within the lesion, remyelinated axons can be distinguished from demyelinated axons because the former have myelin sheaths recognizable as a dark staining rim around the axon. Sections from each animal was examined by an observer blind to the experimental group form which the animal came. The highest rank was given to the animal exhibiting the highest proportion of remyelinated axons. If it was not possible to differentiate two animals using this method then they were given the same rank. In this method, no attempt is made to assign a value to the proportion of remyelination, but simply to establish how a section from an individual animal ranks relative to others. Additional analysis was undertaken by counting the number of remyelinated and demyelinated axons within the lesion by electron microscopy, where remyelinated axons can be identified using the same morphological criteria used in semi-thin section light microscopy. For electron microscopy (EM), ultrathin sections of lesion sites were cut and transferred onto copper grids. The sections were stained with uranyl acetate and imaging was performed using a Hitachi-H600 Transmission Electron Microscope.

G ratio was measured with open source software Fiji (ImageJ, https://imagej.net/Fiji) on transverse electron micrographs at 4,000 – 6,500 magnification with internal calibration. The perimeters of each axon and the myelin sheath were measured with freehand tool on Fiji by tracing the outer surfaces of each structure, then converted the perimeters into hypothetical diameters assuming their circular morphology. The G ratio was calculated as the ratio of the diameter of axon over that of myelin on the same axons, which is inversely correlated to myelin thickness, that a maximum G ratio of 1 results from unmyelinated or demyelinated axons.

#### Isolation of adult oligodendrocyte progenitor cells

Adult male and female rats (2-24 months) were decapitated after lethal injection with phenobarbital. The brains were removed quickly and placed into ice-cold isolation medium (Table S3; alternatively Hibernate A Brainbits). The telencephalon and cerebellum were dissected in isolation medium; meninges, and the olfactory bulb were mechanically removed and the brain tissue was mechanically minced into 1mm^3^ pieces. The tissue pieces were spun down at 100 g for 1min at RT and the tissue was washed in HBSS- (no Mg2+ and Ca^2+^, GIBCO). Each half of the brain was mixed with 5ml of dissociation solution (34U/ml papain (Worthington), 20 μg/ml DNase Type IV (GIBCO) in isolation medium). The brain tissue was dissociated on a shaker (50rpm) for 40 min at 35°C. The digestion was stopped by addition of ice cold HBSS-. The tissue was centrifuged (200 g, 3 min, RT), the supernatant completely aspirated and the tissue resuspended in isolation medium supplemented with 2% B27 and 2mM sodium-pyruvate (trituration solution). The tissue was allowed to sit in this solution for 5min. To obtain a single cell suspension the tissue suspension was triturated 10 times using first a 5ml serological pipette and subsequently three fire polished glass pipettes (opening diameter > 0.5mm). After each trituration step the tissue suspension was allowed to sediment (approximately 1-2 min) and the supernatant (approximately 2ml), containing the cells, was transferred into a fresh tube. After each round of trituration 2ml of fresh trituration solution were added. To remove accidentally transferred undigested tissue bits, the collected supernatant was filtered through 70 μm cell strainers into tubes that contained 90% isotonic Percoll (GE Healthcare, 17-0891-01, in 10xPBS pH7.2 (Lifetech). The final volume was topped up with phenol-red free DMEM/F12 with HEPES (GIBCO) and mixed to yield a homogeneous suspension with a final Percoll concentration of 22.5%. The single cell suspension was separated from remaining debris particles by gradient density centrifugation (800 g, 20min, RT, without break). The myelin debris and all layers without cells were discarded and the brain cell containing phase (last 2ml) and cell pellet were resuspended in HBSS+ and combined in a fresh 15ml tubes and centrifuged (300 g, 5min, RT). The cell pellet was resuspended in red blood cell lysis buffer (Sigma, R7757) and incubated for 1min at RT to remove red blood cells. 10ml of HBSS+ were added to this cell suspension and spun down (300 g, 5min, RT). The cell pellets were resuspended in 0.5ml modified Milteny washing buffer (MWB, 2mM EDTA, 2mM Na-Pyruvate, 0.5% BSA in PBS, pH 7.3) supplemented with 10ng/ml human recombinant insulin (GIBCO). To this cell suspension 2.5μg mouse-anti-rat-A2B5-IgM antibody (Millipore; Table S4) were added for every 10 million cells. After 25 min incubation, gently shaking at 4°C, 7ml of MWB were added. The solution was centrifuged (300 g, 5min, RT) and the pellet resuspended in 80 μL MWB supplemented with 20 μL rat-anti-mouse-IgM antibody (Milteny, 130-047-302) per 10 million cells. The cells were incubated for 15 min, slowly shaking at 4°C. The secondary antibody was again washed out with 7ml MWB and the sample was centrifuged (300 g, 5min, RT). The cell pellet was resuspended in 0.5ml and MACS was performed according to the recommendations of the supplier. Briefly, a MS column (Milteny, 130-042-201) were inserted into MiniMACS Separator (Miltenyi; 130-042-102) and pre-wet with 0.5ml MWB. Resuspended cells were put onto one MS column. Subsequently the column was washed three times using 500 μL MWB for each wash. Finally, A2B5 positive cells were flushed out the column with 1ml pre-warmed, CO_2_ and O_2_ pre-equilibrated OPC medium.

#### Culture of adult oligodendrocyte progenitor cells

Isolated OPCs were seeded onto 12mm glass coverslips in 24 well plates (VWR) or into 96 well-plates (InVitro-Sciences) coated with PDL (Sigma). After isolation, OPCs were left to recover in OPC medium (60μg/ml N-Acetyl cysteine (Sigma), 10μg/ml human recombinant insulin (GIBCO), 1mM sodium pyruvate (GIBCO), 50μg/ml apo-transferrin (Sigma), 16.1μg/ml putrescine (Sigma), 40ng/ml sodium selenite (Sigma), 60ng/ml progesterone (Sigma), 330μg/ml bovine serum albumin (Sigma)) supplemented with b-FGF and PDGF (30ng/ml each, Peprotech). OPCs were incubated at 37°C, 5% CO_2_ and 5% O_2_. The medium was completely exchanged to OPC medium with 20ng/ml bFGF and PDGF after overnight culture to remove any dead cells. After 3d the cell culture medium was switched to promote further proliferation (OPC medium+20ng/ml bFGF and PDGF) or differentiation (OPCM + 40ng/ml T3). During differentiation or proliferation experiments 66% of the medium were replaced every 48h and growth factors or other small molecules were added fresh to the culture. The culture medium used was 500 μL for cultures in 24 well plate wells and 150 μL for cultures in 96 well plate wells. For differentiation assays the medium was in some instances supplemented with 40ng/ml thyroid-hormone (T3, Sigma), 50nM 9-*cis* retinoic acid (9cRA, Sigma), 1μM miconazole (Sigma, M3512) or 1.5μM benztropine (Sigma, SML0847). Otherwise used small molecules: 100μM metformin (Sigma, PHR1084-500MG), rotenone (Sigma, R8875), 1μM dorsomorphin (LC Laboratories, D-3197).

#### Immunofluorescence for tissue sections

Rats received a lethal dose of pento-barbitol and were transcardially perfused with 4% paraformaldehyde (PFA) in PBS. The brains were removed and post-fixed for 2h at RT with 4% PFA. After a rinse in PBS the tissue was incubated in 20% sucrose solution (in PBS) overnight. The tissue was then imbedded in OCT- medium (TissueTek) and stored at −80°C. 12 μm sections were obtained using a cryostat. Tissue sections were air-dried and stored at −80°C. Cryostat cut sections were dried for 45 min at RT. For antigen-retrieval the slides were submerged in preheated citrate buffer pH 6.0 (Sigma) in a water bath at 95°C for 15 min. The slides were washed three times with PBS (5min, RT) and blocked in 0.3% PBST with 10%NDS for 1h at RT. Primary antibodies (Table S4) were diluted in 0.1% PBST with 5%NDS and incubated overnight at 4°C. The slides were washed 3 times for 10min with PBS. Next, secondary antibodies in blocking solution were applied at a concentration of 1:500 for 2h at RT. Slides were washed 3 times with PBS for 10 min each, whereby the first wash contained Hoechst 33342 nuclear stain (2 μg/ml,). The slides were mounted with coverslips using FluoSave (CalBiochem). Image acquisition was performed using a Leica-SP5 microscope (Leica) and LAS software (Leica) or a Zeiss Observer A1 inverted microscope (Zeiss) and Zeiss Axivision software. Further image processing and analysis was performed using the ImageJ software package (Schindelin et al., 2012).

#### Immunofluorescence for cells

Cultured cells were rinsed with PBS before fixation with 4% PFA (10 min, RT). Subsequently, the cells were washed three times with PBS (5 min, RT, shaking). If permeabilisation was required, the cells were incubated with PBST (0.1% Triton X-100 in PBS) for 20 min at RT. The samples were then blocked in PBS supplemented with 10% normal donkey serum (NDS). Primary antibodies (Table S4) were diluted in PBS with 5% NDS and incubated overnight at 4◦C in a humidified chamber. Excess antibodies were washed off with three washes in PBS (10 min, RT, shaking). The primary antibodies were then labeled with secondary antibodies (Table S4) diluted in PBS with 5% normal donkey serum. Again, excess antibody was washed off with three washes PBS (10 min, RT, shaking). If visualization of nuclei was required the first wash contained 2 μg/ml Hoechst 33342 (Sigma). If coverslips were used, they were mounted onto Polysine glass slides (VWR) in a drop of Fluosave (Calbiochem) and the slides were dried for at least 3h at RT in the dark. Images were taken with a Axio-Vision (Zeiss), Leica-SP5 (Leica) or Nikon microscope. For 96 well plate assays cells were kept in PBS after staining. Further image processing and analysis was performed using the ImageJ software package (Schindelin et al., 2012).

#### Comet assay

For comet assays, a single cell gel electrophoresis based assay for detecting DNA damage, approximately 5000 OPCs were resuspended in 100μl PBS and mixed with 300 μl 1% low melting point agarose (37°C). Alternatively, when OPCs were cultured prior to the assay, the cells were detached using TrypLE 1x Select (GIBCO) for 8 min at 37°C. The comet assay was then performed as described in Olive and Banáth (2006). Briefly, OPCs were centrifuged at 300 g for 5 min. at room temperature and the cell pellet was resuspended with 100μl PBS and then mixed with 300μl molten low-melting point agarose pre-incubated at 37°C. The cell-agarose suspension was then applied gently onto polysine slides that were pre-treated with 1% agarose and allowed to solidify at 4°C. The slides were submersed in alkaline cell lysis buffer (0.3M NaOH, 100mM EDTA, 0.1% (w/v) N-Lauroylsarcosine (Sigma, 61745), 1.2M NaCl in ddH2O) for 16 h at 4°C in the dark. The slides were then electrophoresed in alkaline electrophoresis buffer (0.03M NaOH, 2mM EDTA, pH > 12.3, pre-chilled at 4°C) for 25 min at RT with 1V/cm, whereby *cm* represents the distance between the electrodes. Finally, electrophoresed and propidium iodide stained DNA was visualized using a Zeiss Axiovision Fluorescence microscope (Carl Zeiss), and 50-100 nuclei per animal were visually scored according to published protocols (Collins, 2004). Statistical significance was determined comparing respective damage categories between experimental groups by a two-tailed unpaired t test. A significant result was assumed for p < 0.05.

#### RNA sequencing and downstream analysis

RNA was isolated from freshly purified young adult (2-3 months) and aged (20-24 months) OPCs using QIAGEN RNAeasy Micro kit (QIAGEN) and RNA was stored at −80°C. RNA quality was assessed by Qubit measurement and posterior RNA nanochip/picochip Bioanalyzer. Ribosomal RNA was depleted with rat-specific oligos (InDA-C technology). Sequencing libraries were prepared using 10-100ng total RNA and the Nugen Ovation RNA-Seq Systems 1–16 for Model Organisms Kit (0349-32). Sequencing was performed on the Illumina HiSeq4000 in a pair-end 150 base pair format. Adaptor sequences were removed and reads were quality-trimmed using TrimGalore. Trimmed reads were aligned to the rat reference genome (RGSC6.0/rn6) by using TopHat2 (Kim et al., 2013) (http://ccb.jhu.edu/software/tophat, version: 2.0.13) guided by Ensembl gene models. Raw counts per gene regions were obtained by *featureCounts*. Replicates were evaluated, counts were normalized and differential expression of transcripts was evaluated by the R Bioconductor *DESeq2* package (Love et al., 2014). Expression levels were further normalized by transcript length (per kB). Transcript annotations were based on Ensembl (Release 82). GO term analysis was performed using the *goseq* package. For ingenuity pathway analysis we used differentially expressed genes with an adjusted p value cutoff (p.adj < 0.05). The OPC gene dataset for Figure 2A was taken from Table S1 from Marques et al. (2016).

Raw data files from RNaseq were made available at the NCBI GEO database (accession number GSE134765).

#### RNA isolation and qRT-PCR

RNA was isolated from freshly purified OPCs or from cultured OPCs according to the Directzol RNA MicroPrep Kit (Zymo Research; R2061). All RNA samples were stored at −80°C prior to further processing. cDNA was generated using the QuantiTect Reverse Transcription Kit’s according to the instructions of the manufacturer (QIAGEN; 205310). For RT-qPCR, primers (see Table S5) were used at a concentration of 400μM. The efficiency of each primer was greater than ∼95% as determined for each primer pair by serial dilutions of OPC cDNA. cDNA, primers, and the Syber Green Master Mix (QIAGEN; 204141) were mixed as instructed by the manufacturer, and RT-qPCR and melting curve analysis were performed on Life Technologies’ Quantstudio 6 Flex Real-Time PCR System. Fold changes in gene expression were calculated using the delta delta Ct method in Microsoft Excel. Statistical significance was determined using two-tailed unpaired t tests assuming equal variances.

#### ATP measurements

For the comparison of freshly isolated cell populations (young, aged and ADF aged) cells were spun down (300 g, 5min). The supernatant was removed and the cell pellets were frozen down at −80°C and stored until analysis. The relative ATP content was measured using the ATPlite luminescence Assay System (Perkin Elmer). Cell pellets were resuspended in 100μl OPC medium, alternatively freshly cultured cells in 100μl OPC medium were used. To this suspension 100μl of lysis buffer were added and the samples shaken at 600rpm for 5min on a horizontal shaker. 50μl of substrate solution were added and the samples were shaken at 600rpm in a horizontal shaker for another 5min. The luminescence signal was recorded using an Infinite pro 200 Tecan plate reader (Tecan) and the Magellan software. The luminescence was measured for 1 s intervals. The counts were normalized to cell numbers.

#### Oxygen Consumption rate measurements

OPCs or pre-oligodendrcoytes were seeded onto PDL coated Seahorse cell culture plates (Agilent). The oxygen consumption rate was recorded using the manufacturers standard protocol for mitochondrial stress tests (Agilent). For the comparison of young and aged cells or OPCs and POLs, the cells were cultured overnight prior to the assay. For the comparison of aged cells treated with metformin and dorsomorphin the cells were cultured for 5d in the presence of the drugs. The cells were cultured 1h before the assay in modified assay medium (1.5mM sodium pyruvate, 2mM L-Glutamine, 2mM Glucose, 1% SATO in XF base medium, pH 7.4). The OCR measurements were carried out using a Seahorse XFp or a Seahorse XF96 analyzer. The basal OCR was calculated as the difference between the average of the measurements taken under untreated conditions and the average of the measurements taken after the injection of rotenone and antimycin A. All OCR values were then normalized to the cell number. The final OCR values were normalized to one treatment group and the results are represented as the relative basal oxygen consumption rate between the groups. The concentrations of the small molecules in the assay were: oligomycin (1μM), FCCP (0.5μM) rotenone (0.5μM) and antimycin A (0.5μM)

#### Western Blot

Cells were lysed in IP lysis buffer (Thermo Scientific) supplemented with 1% Halt protease inhibitor (Thermo Scientific, 87786) for 10 min on ice. The lysates were spun down for 10min at 4°C and 10, 000 g in a table top centrifuge. The supernatant was stored at −80°C. Protein quantification was carried out using Pierce BCA protein assay kit (Thermo Scientific) measured with a Nanodrop2000. Equal amounts of protein (15-20μg) were loaded mixed with 4X Bolt® LDS Sample Buffer (Thermo Fisher; B0007) and 10X Bolt reduction agent and boiled to 70°C for 10min. Protein was run on Bolt 4%–12% Bis-Tris Plus Gels (Thermo Fisher; NW04120BOX) in Bolt MOPS SDS running buffer (Thermo Scientific, B0001) for 32min at 200V. Protein was transferred for 60min at 20V to a nitrocellulose membrane (Immobilon FL 0.45μm pore size, Millipore) membrane using the Mini Blot module (Thermo Scientific, B1000) and Bolt transfer buffer with 10% methanol and 1% Bolt antioxidant (Thermo Scientific, BT005) according to the manufacturer’s instructions. Membranes were blocked in 50% Odyseey blocking buffer TBS (Li-Cor, 927-50100) in TBS. All primary antibodies were used in a dilution of 1:1000 in 0.1%TBS-Tween with 50% Odyseey blocking buffer (TBS) in TBS. The membranes were incubated shaking in antibody solution overnight at 4°C. The membranes were washed twice in 0.1% TBS-Tween. Secondary antibodies were added in a concentration of 1:10000 in 50% Odyssey blocking buffer in 0.1% TBS-Tween. Secondary antibodies were incubated at room temperature for 1h in the dark. The membranes were washed three times in 0.1% TBS Tween. Fluorescent antibody signal was detected using the Odyssey (Li-Cor) and Image Studio v4.0 software. For luminescent signal detection the membranes were incubated with ECL solution (Amersham ECL Western Blot analysis system, GE Healthcare) and the signal was detected as described for fluorescent signals. For PGC1a detection we used a secondary antibody anti-mouse-IgG coupled to HRP (CST) and the

#### Fluorescence Activated Cell Sorting

Freshly isolated OPCs were fixed in ice-cold 4% PFA for 10min and washed in FACS buffer (0.5% BSA in PBS). The cells were stained with primary antibodies (Anti-A2B5-PE, anti-MOG-biotin and anti-rat-Cd11b-PerCP-Cy5.5 and appropriate isotype controls; see Table S4) for 30min at 4°C. With exception of A2B5 staining, which was carried out for 10min at 4°C. Cells were washed with FACS buffer and stained with secondary antibody (Streptavidin-BV421) for 15min at 4°C in the dark. Cells were washed and resuspended in FACS buffer. Cells were analyzed using an Attune-NXT (Thermo Scientific) equipped with 405, 488 and 561 lasers. For compensation, beads (OneComp) were used for single stains for each fluorophore. The compensation matrix was automatically calculated and applied by the Attune software. Gates for the quantification of A2B5, Cd11b and MOG positive cells were set according to appropriate FMOs. A minimum of 30,000 cell singlets were recorded and used for quantification with FlowJo software (v10).

#### Oil-Red-O staining

12μm cryostat cut sections were dried for 45 min at RT. Tissue sections were dehydrated in 100% 1,2 propanediol (Sigma) for 2 times 5 min. The slides were stained at 60C in prewarmed 0.5% Oil Red O solution (ORO, Sigma). Then, the samples were placed into 85% 1,2 propanediol (v/v in distilled water) for 8 min to differentiate the staining. The slides were then rinsed three times with distilled water and mounted with jelly mounting media. Image acquisition was performed with a Nikon microscope. Digital images were converted into 8-bit greyscale images. The pixel values were inverted so that a more intense staining corresponded to higher pixel values. The staining was quantified using ImageJ software, measuring the mean gray value for the lesion area.

#### CRISPR/Cas9 mediated knockdown of Prkaa2 (AMPK)

Using Phusion polymerase (Thermo Fisher; F530S) a T7 promoter Cas9 PCR product was amplified from the AAV-CMVc-Cas9 plasmid (Addgene plasmid # 106431, Juan Ipiszua Belmonte’s laboratory). The product was purified and used as a template to generate capped mRNA using the HiScribe mRNA kit with tailing (NEB, E2060S). All guide RNAs were designed using CHOPCHOP (https://chopchop.cbu.uib.no). DNA Templates containing a T7 promoter site for the generation of non-targeting and prkaa2 gRNAs were assembled by oligo annealing to a gRNA scaffold sequence using Klenow fragment (NEB, M0210S). gRNAs were then produced by *in vitro* transcription using T7 polymerase (NEB, M0251S). gRNAs were purified using RNA extraction columns (Zymo, R2060). Capped Cas9 mRNA and gRNAs were transfected into aged OPCs (seeded at 30,000 cells per 96 well to achieve approximately 80%–90% confluency) using Lipofectamine LTX (Thermo Scientific, 15338100, 0.4μl LTX, 0.1μl Plus reagent, 100ng Cas9 mRNA, 50ng gRNAs per 96 well in Opti-MEM, final volume 10μl). The medium was completely replaced after 16h.

### Quantification and Statistical Analysis

#### Statistical analysis

All statistical analysis was performed in GraphPad Prism (GraphPad Software, Inc.) or R. Prior to the analysis of parametric data we performed Shapiro-Wilk tests to ensure normality of the data. For data derived from the quantification of immunohistochemical staining, comparisons between two groups were performed with an unpaired t test assuming two-tailed distribution and equal variances. In cases where more than two groups were compared to each other, a one-way analysis of variance (ANOVA) was performed assuming equal variances, followed by an appropriate post-test to compare individual groups. For ranking analysis of remyelination, the non-parametric Mann-Whitney test was used to determine whether two groups differed in their extent of remyelination. A comparison of the extent of remyelination between three groups was performed using a Kruskal-Wallis test followed by Dunn’s post-test to compare individual groups. For qRT PCR data two groups were compared to each other using unpaired two-tailed t test. For data derived from comet assays damage categories would be tested between treatment groups using unpaired two-tailed t tests. For all statistical tests, differences were considered significant at p < 0.05.

### Data and Code Availability

The RNA sequencing data of young and aged oligodendrocyte progenitor cells (OPCs) generated during this study are available at GEO: GSE134765.

## Supplemental Information

Document S1. Figures S1–S6 and Tables S3–S5

Table S1. Log2-Transformed RNA-Seq Expression Data for Characteristic OPC Genes between Young and Aged OPCs, Related to Figure 2A

Table S2. Ingenuity Pathway Analysis Results for Genes Significantly More Highly Expressed in Aged versus Young OPCs, Related to Figure 2C

Document S2. Article plus Supplemental Information
