# Supplementary material for: Metformin Restores CNS Remyelination Capacity by Rejuvenating Aged Stem Cells
Source: Cell Stem Cell. 2019 Oct 3;25(4):473–485.e8. doi: 10.1016/j.stem.2019.08.015 (PMC6863391; doi:10.1016/j.stem.2019.08.015)
Supplement: Document S1. Figures S1–S6 and Tables S3–S5 [file mmc1.pdf]

Cell Stem Cell, Volume *25*

Supplemental Information

Metformin Restores CNS Remyelination Capacity

by Rejuvenating Aged Stem Cells

Björn Neumann, Roey Baror, Chao Zhao, Michael Segel, Sabine Dietmann, Khalil S. Rawji, Sarah Foerster, Crystal R. McClain, Kevin Chalut, Peter van Wijngaarden, and Robin J.M. Franklin

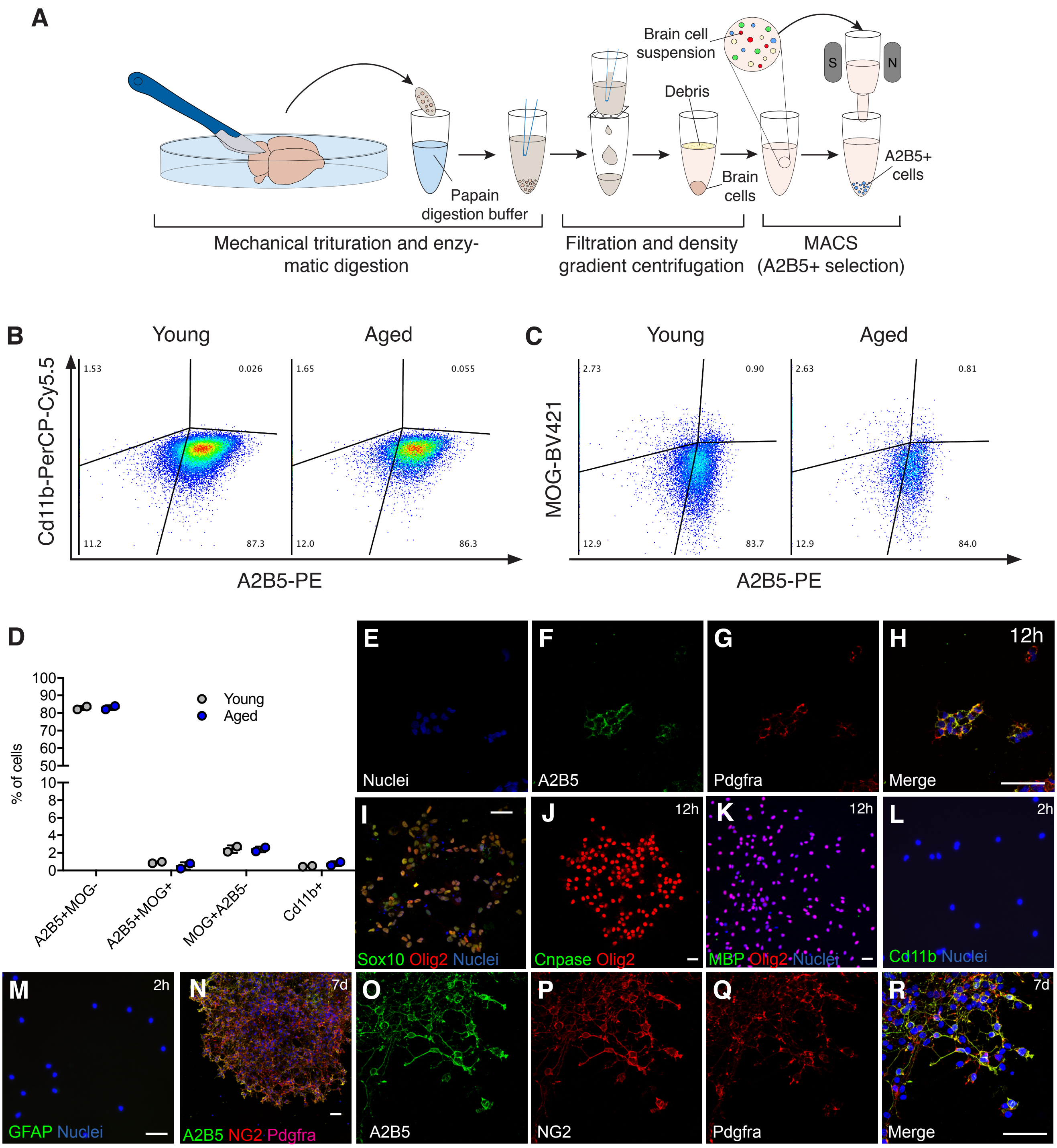

A
Brain cell suspension
S
N
Debris
Brain cells
A2B5+ cells
Papain digestion buffer
Mechanical trituration and enzy-matic digestion
Filtration and density gradient centrifugation
MACS (A2B5+ selection)
B
Young
Aged
Cd11b-PerCP-Cy5.5
A2B5-PE
C
MOG-BV421
D
% of cells
Young
Aged
A2B5+MOG-
A2B5+MOG+
MOG+A2B5-
Cd11b+
E
Nuclei
F
A2B5
G
Pdgfra
H
12h
Merge
I
Sox10 Olig2 Nuclei
J
12h
Cnpase Olig2
K
12h
MBP Olig2 Nuclei
L
2h
Cd11b Nuclei
M
2h
GFAP Nuclei
N
7d
A2B5 NG2 Pdgfra
O
A2B5
P
NG2
Q
Pdgfra
R
7d
Merge

Figure S.1. Related to Figure 1. Isolation and culture of adult OPCs from aged rats. (A), Schematic illustration of the adult OPC isolation workflow. (**B-C),** Representative density plots of FACS data from freshly isolated cells stained for A2B5 (OPC marker) and Cd11b (microglia marker) **(B),** and A2B5 and MOG (oligodendrocyte marker) (**C**). **(D),** Quantification of FACS data presented in B and C (n=2 biological repeats). **(E-H),** A2B5^{+} sorted cells are positive for OPC markers A2B5 and Pdgfra. **(I),** More than 90% of A2B5 cells are positive for Sox10 and Olig2. **(J-K),** A2B5 sorted cells do not express more mature lineage markers such as CNPase or MBP. (**L-M)**, The A2B5^{+} population does not contain significant contamination from other glia cells such as Cd11b^{+} microglia **(L)**, or GFAP^{+} astrocytes **(M)**. **(N),** In the presence of PDGF-AA and b-FGF, A2B5^{+} isolated cells form colonies of mostly bipolar cells that express the OPC markers A2B5, NG2 and Pdgfra. **(O-R)**, Higher magnification of A2B5, NG2, Pdgfra cells. Images in E-Q are taken from cultures of aged OPCs (≥18 months). Scale bar = 50µm.

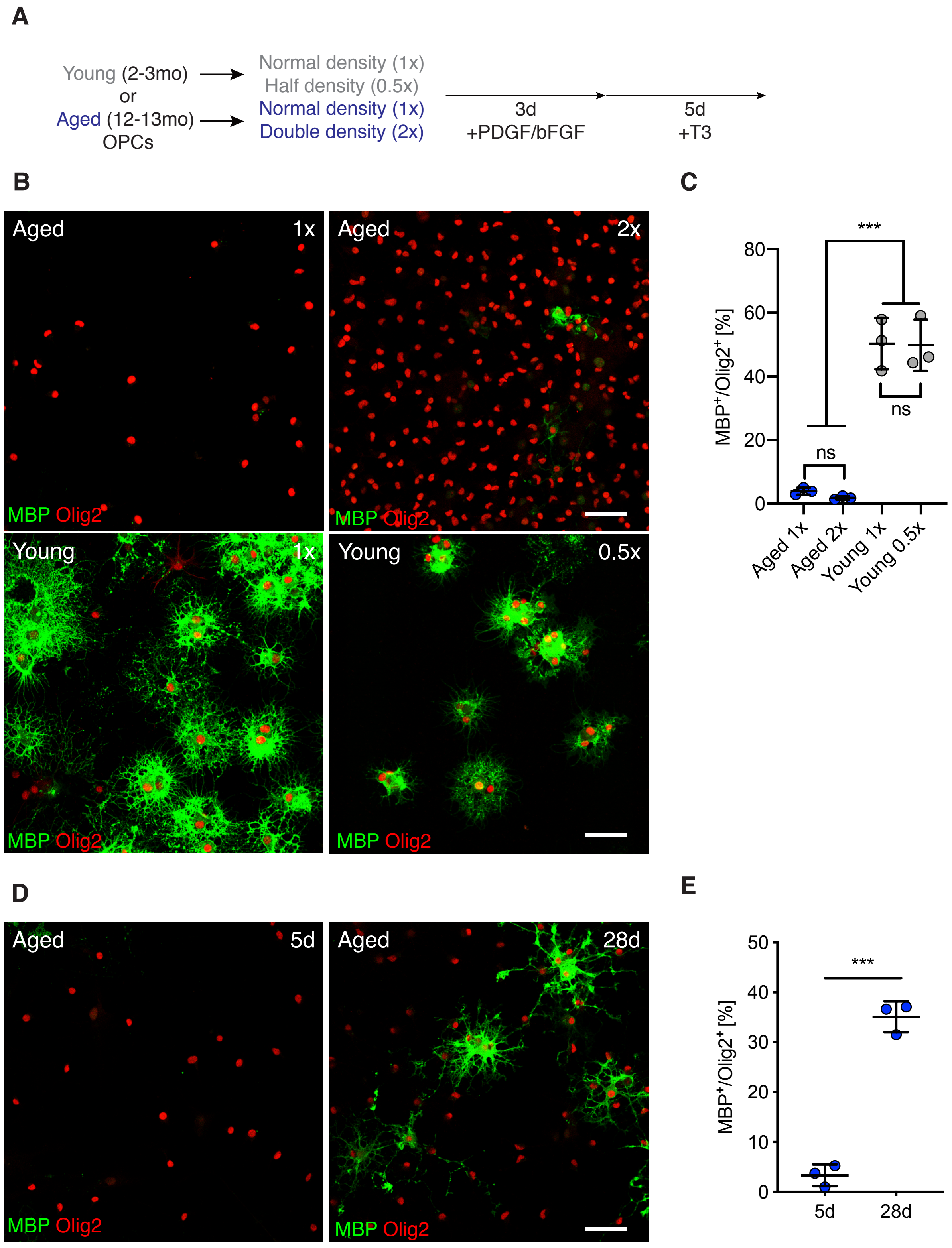
A
Young (2-3mo)
or
Aged (12-13mo)
OPCs
Normal density (1x)
Half density (0.5x)
Normal density (1x)
Double density (2x)
3d
+PDGF/bFGF
5d
+T3
B
Aged 1x
Aged 2x
Young 1x
Young 0.5x
MBP Olig2
C
MBP+/Olig2+ [%]
***
ns
ns
Aged 1x
Aged 2x
Young 1x
Young 0.5x
D
Aged 5d
Aged 28d
MBP Olig2
E
MBP+/Olig2+ [%]
***
5d
28d

Figure S.2. Related to Figure 1. Density changes do not alter the differentiation rate of OPCs. **(A)** Schematic of the experimental design. Young (2-3 months old) or aged OPCs (18-20 months) were recovered in growth factor-supplemented (PDGF-AA and b-FGF) medium for 3d and were then subjected to pro-differentiation compounds for 5d. Young OPCs were seeded in the regular or half the density. Aged OPCs were seeded in the regular or double density. **(B),** Representative images of the differentiation assay. Newly formed oligodendrocytes were identified as $Olig2^{+}MBP^{+}$ cells. **(C)**, Quantification of the differentiation assay. All data are presented as mean ± SD (n=3 biological replicates, One Way ANOVA with Dunnett's multiple comparison test for each group against each other). **(D)** Aged OPCs (18-20 months) were cultured as described above. Differentiation was assessed 5d or 28d after removal of growth factors and addition of T3. Newly formed oligodendrocytes were identified as $Olig2^{+}MBP^{+}$ cells. **(E)** Quantification of the differentiation assay. All data are presented as mean ± SD (n=3 biological replicates, two-tailed t-test). All scale bars = 50µm. *P<0.05, **P<0.01, ***P<0.001.

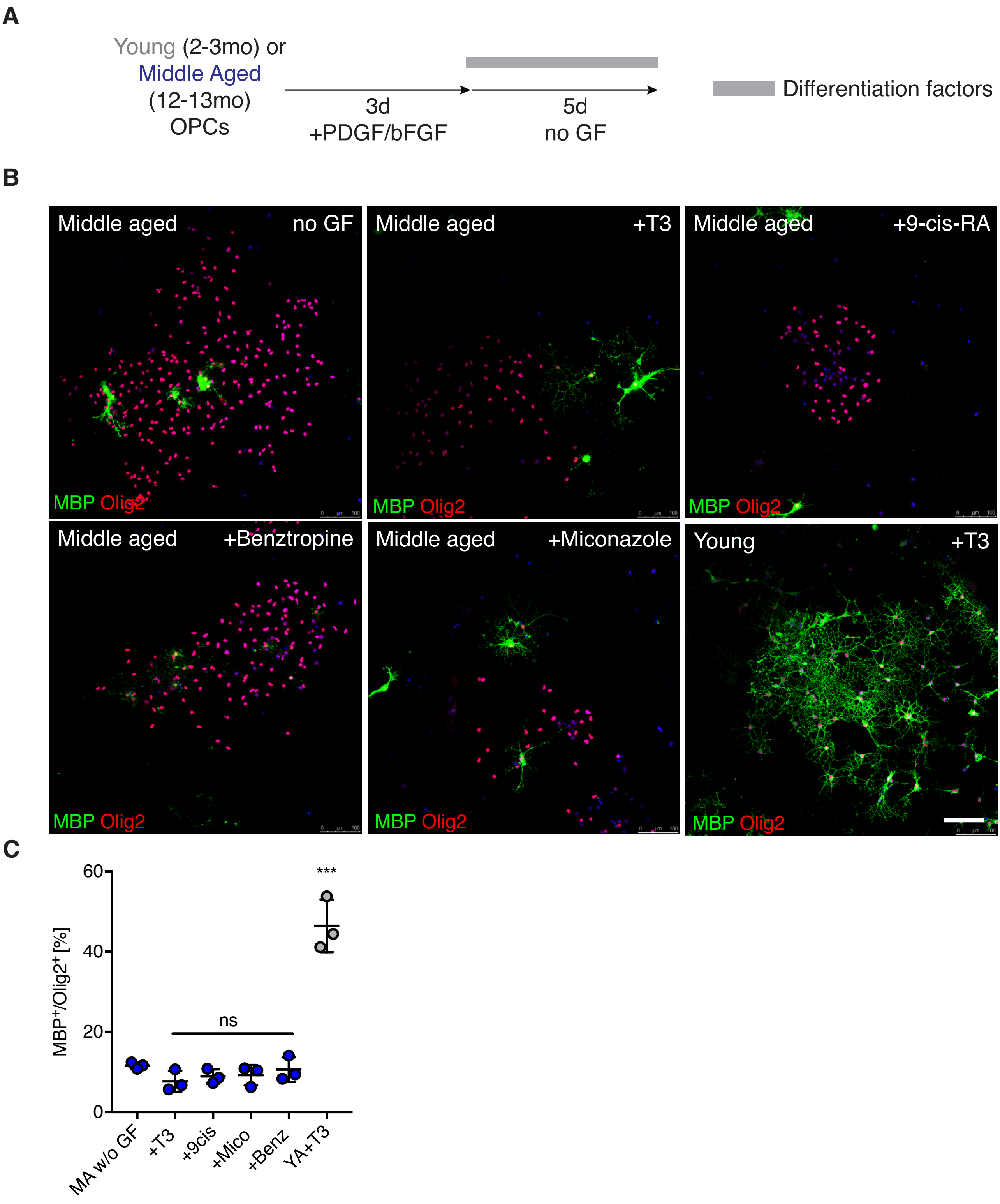
A
Young (2-3mo) or
Middle Aged
(12-13mo)
OPCs
3d
+PDGF/bFGF
5d
no GF
Differentiation factors
B
Middle aged no GF
Middle aged +T3
Middle aged +9-cis-RA
Middle aged +Benztropine
Middle aged +Miconazole
Young +T3
MBP Olig2
C
MBP+/Olig2+ [%]
60
40
20
0
ns
***
MA w/o GF
+T3
+9cis
+Mico
+Benz
YA+T3

Figure S.3. Related to Figure 1 and 3. Middle aged OPCs lose their inherent differentiation potential and responsiveness to pro-differentiation compounds. (A), Schematic of the experimental design. Young (2-3 months old) or middle aged OPCs (12-13 months old) were recovered in growth factor-supplemented (PDGF-AA and b-FGF) medium for 3d and were then subjected to pro-differentiation compounds for 5d. **(B),** Representative images of the differentiation assay. Newly formed oligodendrocytes were identified as $Olig2^{+}MBP^{+}$ cells. Scale bar = 50µm. (**C**), Quantification of the differentiation assay. All data are presented as mean ± SD (n=3 biological replicates, One Way ANOVA with Dunnett's multiple comparison test for each group against the middle aged group differentiating in the absence of growth factors “MA”). *P<0.05, **P<0.01, ***P<0.001.

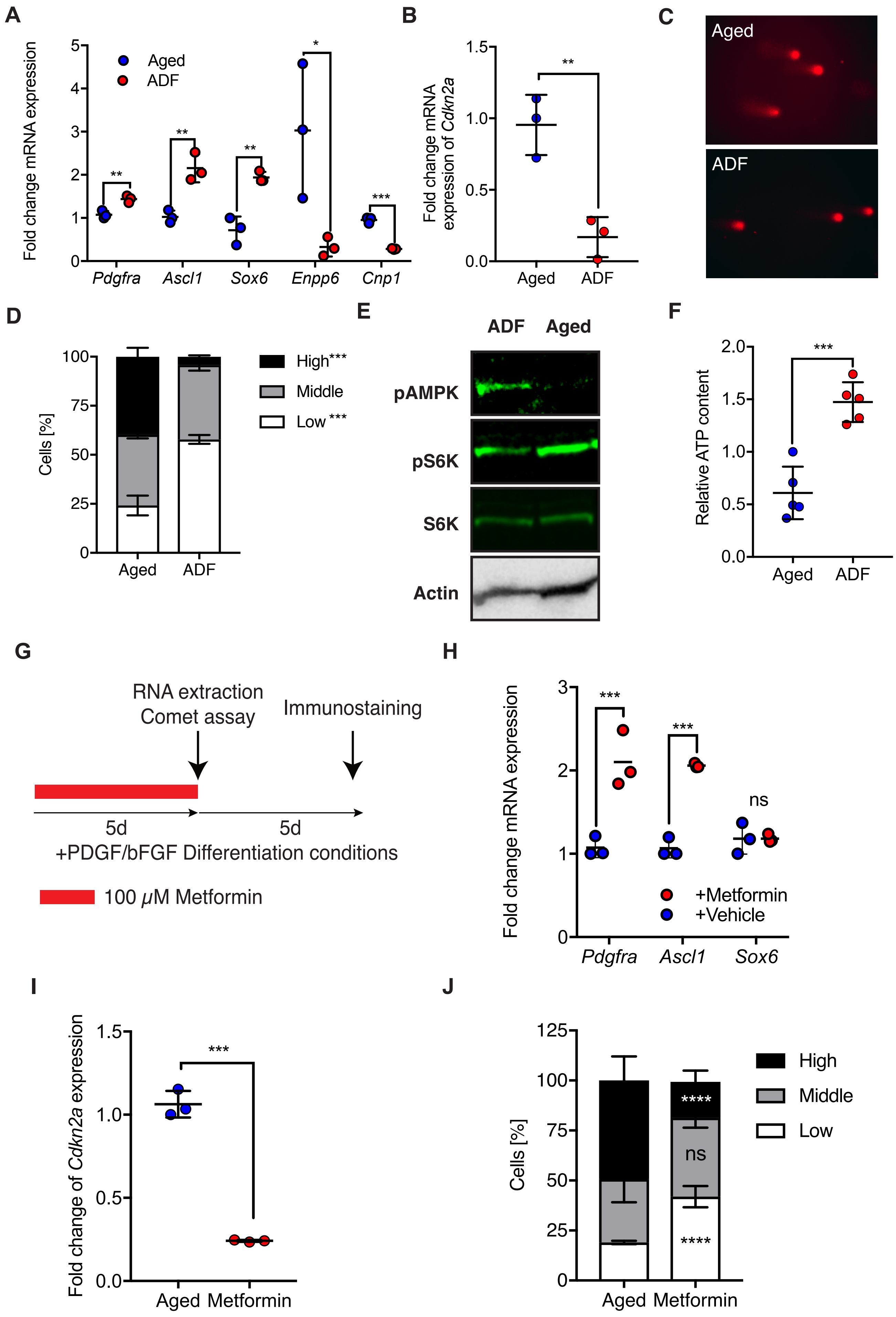

A
Fold change mRNA expression
Aged
ADF
Pdgfra
Ascl1
Sox6
Enpp6
Cnp1
B
Fold change mRNA expression of Cdkn2a
Aged
ADF
C
Aged
ADF
D
Cells [%]
High***
Middle
Low***
Aged
ADF
E
ADF
Aged
pAMPK
pS6K
S6K
Actin
F
Relative ATP content
Aged
ADF
G
RNA extraction
Comet assay
Immunostaining
5d
5d
+PDGF/bFGF Differentiation conditions
100 μM Metformin
H
Fold change mRNA expression
ns
+Metformin
+Vehicle
Pdgfra
Ascl1
Sox6
I
Fold change of Cdkn2a expression
Aged
Metformin
J
Cells [%]
High
Middle
Low
ns
Aged
Metformin

Figure S.4. Related to Figure 3 and 4. ADF and treatment of aged OPCs with metformin in vitro reverses hallmarks of ageing. Animals were exposed to alternate day fasting or fed *ad libitum* as described in Figure 3. At 18 months of age OPCs were isolated from both experimental groups. **(A),** qRT-PCR for OPC genes and **(B),** Cdkn2a. All data are presented as mean ± SD (n=3 biological replicates, two-tailed t-test). **(C),** Representative images of comet assay data. **(D)**, Quantification of comet assays. All data are presented as mean ± SD (n=3 biological replicates, two tailed t-test comparing each damage category between the two groups, i.e. Aged Low vs ADF Low). **(E),** Representative Western Blot data from acutely isolated OPCs from aged (18 months) and ADF animals (18 months). n=2 biological repeats for each experimental group. **(F),** Quantification of normalised intracellular ATP content (n=5 biological repeats for each experimental group). **(G),** OPCs were isolated from aged animals (≥18 months) and cultured in the presence of growth factors for 5 days. Some cells were treated with 100µM metformin with each medium change during the first 5 days ("metformin" days 2 and 4), whereas control cells ("Aged") received medium alone. **(H),** qRT-PCR for OPC genes (*Pdgfra*, *Ascl1* and *Sox6*) and **(I**), Cdkn2a. All data are presented as mean ± SD (n=3 biological replicates for each group, two-tailed t-test). **(J),** Quantification of comet assays. All data are presented as mean ± SD (n=3 biological replicates for each group, two-tailed t-test comparing each damage category between the two experimental groups, i.e Aged Low and Metformin low). *P < 0.05, **P<0.01, ***P<0.001, ns P>0.05

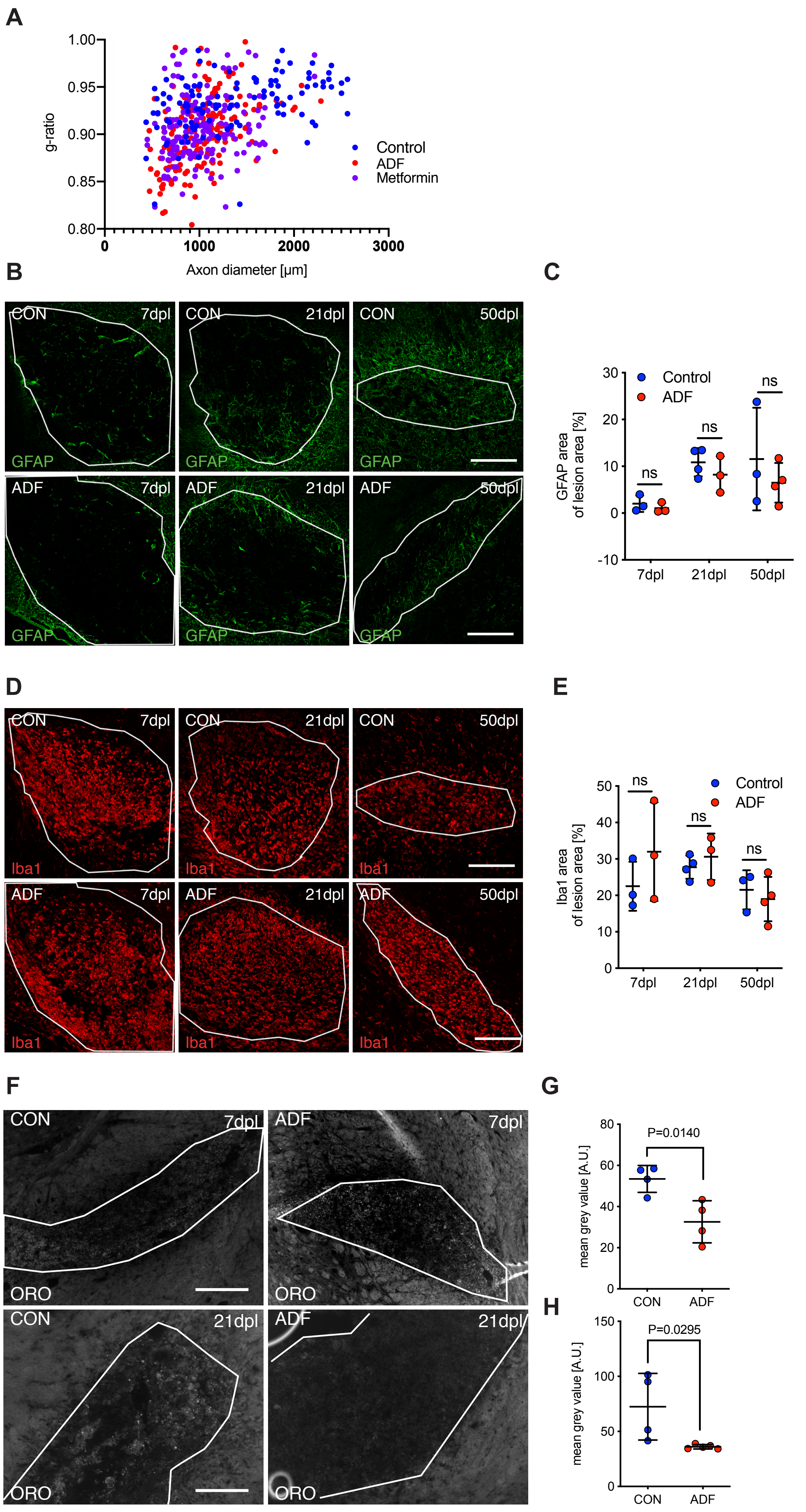
A
g-ratio
1.00
0.95
0.90
0.85
0.80
0
1000
2000
3000
Axon diameter [μm]
Control
ADF
Metformin
B
CON 7dpl
CON 21dpl
CON 50dpl
ADF 7dpl
ADF 21dpl
ADF 50dpl
GFAP
C
GFAP area of lesion area [%]
Control
ADF
ns
30
20
10
0
-10
7dpl
21dpl
50dpl
D
CON 7dpl
CON 21dpl
CON 50dpl
ADF 7dpl
ADF 21dpl
ADF 50dpl
Iba1
E
Iba1 area of lesion area [%]
Control
ADF
ns
50
40
30
20
10
0
7dpl
21dpl
50dpl
F
CON 7dpl
ADF 7dpl
CON 21dpl
ADF 21dpl
ORO
G
mean grey value [A.U.]
P=0.0140
80
60
40
20
0
CON
ADF
H
mean grey value [A.U.]
P=0.0295
150
100
50
0
CON
ADF

Figure S.5. Related to Figure 3. Additional analysis about remyelinating lesions. (A), G-ratio analysis of ADF, Control and Metformin remyelinated axons. **(B),** Representative images of lesions stained for the reactive astrocyte marker GFAP at 7,21 and 50dpl. **(C),** Quantification of the area within a lesion covered by GFAP. **(D),** Representative images of lesions stained for the macrophage and microglia marker Iba1 at 7,21 and 50dpl. **(E),** Quantification of the area within a lesion covered by Iba1. **(F, H),** Representative images of lesions stained Oil-Red-O to visualise myelin debris at 7dpl (F) and 21dpl (H). **(G, I),** Quantification of the myelin debris load in a lesion at 7dpl (G, n= 4 biological repeats, two-tailed t-test) and 21dpl (I, n=4 biological repeats, two-tailed t-test). All scale bars: 100µm. ns: $P>0.05$

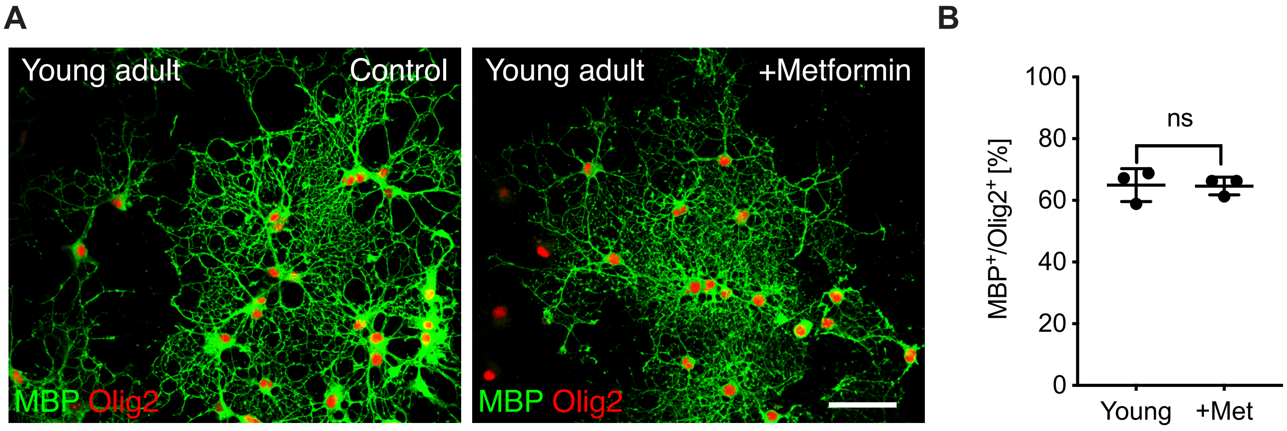
A
Young adult
Control
MBP Olig2
Young adult
+Metformin
MBP Olig2
B
100
80
60
40
20
0
ns
MBP+/Olig2+ [%]
Young
+Met

Figure S.6. Related to Figure 4. Metformin does not accelerate the differentiation of young OPCs. (A) Young OPCs (2-3 months old) were recovered in growth factor-supplemented (PDGF-AA and b-FGF) medium for 3d and were then differentiated for 5d. Metformin treated cells were exposed to metformin throughout the whole culture period. Newly formed oligodendrocytes were identified as $Olig2^{+}MBP^{+}$ cells. **(B)** Quantification of the differentiation assay. All data are presented as mean ± SD (n=3 biological replicates, two-tailed t-test). All scale bars = 50µm. ns: $P>0.05$.

SI Table 1. Log2 transformed RNAseq expression data for characteristic OPC genes between young and aged OPCs. Related to Figure 2A.

SI Table 2. Ingenuity Pathway analysis results for genes significantly higher expressed in aged versus young OPCs. Related to Figure 2C.

SI Table. 3. Formulation of isolation medium. Related to STAR Methods. All chemicals were purchased from Sigma. pH was adjusted to 7.3 and the medium was filtered (0.22µm) and stored at 4ºC.

**Amino Acids**	**µM**	**MW**	**mg/5l**
Glycine	400	75.07	150.1
L-Alanine	22	89.09	9.8
L-Arginine hydrochloride	483	174.2	420.7
L-Asparagine-H2O	5.5	150.13	4.1286
L-Cysteine hydrochloride-H2O	7.7	313.2	12.06
L-Histidine hydrochloride-H2O	200	209.6	209.6
L-Isoleucine	802	131.2	526.1
L-Leucine	802	131.2	526.1
L-Lysine hydrochloride	798	146.2	583.3
L-Methionine	201	149.2	149.9
L-Phenylalanine	400	165.2	330.4
L-Proline	67	115.13	38.569
L-Serine	400	105	210
L-Threonine	798	119	474.8
L-Tryptophan	78	204.2	79.6
L-Tyrosine disodium salt dihydrate	398	181.2	360.6
L-Valine	803	117.2	470.6
**Vitamins**			
Choline chloride	28	139.62	19.55
D-Calcium pantothenate	8	238.27	9.53
Niacinamide	30	122	18.3
Pyridoxine hydrochloride	20	206	20.6
Thiamine hydrochloride	10	337	16.9
i-Inositol	40	180.2	36
**Inorganic Salts**			
Ferric Nitrate (Fe(NO3)3"9H2O)	0.25	404	0.5
Potassium Chloride (KCl)	5360	74.55	1997.9
Sodium Bicarbonate (NaHCO3)	880	84	369.6
Sodium Chloride (NaCl)	89000	58	25810
Sodium Phosphate dibasic (Na2HPO4) anhydrous	906	120	543.6
Zinc sulfate (ZnSO4-7H2O)	0.67	287.56	0.9633
**Other Components**			
D-Glucose (Dextrose)	25000	180.2	22525
Sodium Pyruvate	227	110.04	124.9
MOPS	10000	269.3	13465

SI Table 4. Antibodies used in the study. Related to STAR Methods. AF: Alexa Fluor, PE: Phycoerythrin, BV: Brilliant Violet, SA: Streptavidin

Antibody	Class	Host species	Dilution (FACS)	Dilution (in vitro)	Dilution (in vivo)	Source	Catalogue number
Anti-Olig2	IgG	Rabbit	-	1:1000	1:500	Millipore	AB9610
Anti-Sox10	IgG	Goat	-	1:100	1:100	Santa Cruz	sc-17342
Anti-Pdgfra	IgG	Rat	-	1:300	-	BD Pharmingen	558774
Anti-NG2	IgG	Rabbit	-	1:500	-		MAB5320
Anti-A2B5	IgM	Mouse	-	1:500	-	Millipore	MAB312
Anti-Ki67	IgG	Rabbit	-	-	1:500	Abcam	Ab166667
Anti-CC1 (APC)	IgG	Mouse	-	-	1:300	Calbiochem	OP80
Anti-O4	IgM	Mouse	-	1:1000	-	R&D Systems	PZO
Anti-CNPase	IgG	Mouse	-	1:1000	-	Abcam	Ab6319
Anti-MBP	IgG	Rat	-	1:500	-	Serotec	MCA4095
Anti-Nkx2.2	IgG	Mouse	-	-	1:200	DHSB	745A5
Anti-Cd11b/c	IgG	Mouse	-	1:200	-	Serotec	MCA275R
Anti-GFAP	IgG	Goat	-	1:2000	-	Abcam	Ab53554
Anti-A2B5-PE	IgM	Mouse	1:10	-	-	Milteny	105HB29
Mouse IgM-PE	IgM	Mouse	1:10	-	-	Milteny	IS5-20C4
Anti-MOG-Biotin	IgG	Goat	1:40	-	-	R&D Systems	BAF2439
IgG-biotin Control	IgG	Donkey	1:40	-	-		
Anti-Cd11b-PerCP-Cy5.5	IgG	Mouse	1:1000	-	-	Biolegend	Ox-42
Mouse IgG-PerCP-Cy5.5	IgG	Mouse	1:1000	-	-	Biolegend	MOPC1-73
SA-BV421	-	-	1:800	-	-	Biolegend	405226
Anti-Mouse IgG AF-488		Donkey	-	1:1000	1:500	Invitrogen	A21202
Anti-Mouse IgG AF-594		Donkey	-	1:1000	1:500	Invitrogen	A21203
Anti-Mouse IgM AF-488		Goat	-	1:1000	1:500	Invitrogen	A21042

Anti-Mouse IgG AF-647		Donkey	-	1:1000	1:500	Invitrogen	A31571
Anti-Rabbit IgG AF-488		Donkey	-	1:1000	1:500	Invitrogen	A21206
Anti-Rabbit IgG AF-594		Donkey	-	1:1000	1:500	Invitrogen	A21207
Anti-Rabbit IgG AF-647		Donkey	-	1:1000	1:500	Invitrogen	A21447
Anti-Rat IgG AF-488		Donkey	-	1:1000	1:500	Invitrogen	A21208
Anti-Rat IgG AF-594		Donkey	-	1:1000	1:500	Invitrogen	A21209
Anti-Goat IgG AF-488		Donkey	-	1:1000	1:500	Invitrogen	A11055
Anti-Goat IgG AF-647		Donkey	-	1:1000	1:500	Invitrogen	A21147
**Antbodies for Western Blot**	**Class**	**Host species**	**Dilution (WB)**			**Source**	**Catalogue number**
Anti-AMPK	IgG	Mouse	1:1000			Abcam	Ab80039
Anti-phospho-	IgG	Rabbit	1:1000			CST	2353
AMPK Anti-p70S6k	IgG	Rabbit	1:1000			CST	9202
Anti-phospho-p70S6K	IgG	Rabbit	1:1000			CST	9205
Anti-S6 Ribosomal Proten	IgG	Mouse	1:1000			CST	2317
Anti-phospho-S6	IgG	Rabbit	1:1000			CST	4858
Anti-PGC1a	IgG	Mouse	1:1000			Calbiochem	ST1202
Anti-beta-actin-HRP	IgG	Mouse	1:20000			Sigma	A3854
IRDye680 LT anti-mouse	IgG	Donkey	1:10000			Li-Cor	926_68022
IRDye680 LT anti-rabbit	IgG	Donkey	1:10000			Li-Cor	926_38073
IRDye800CW	IgG	Donkey	1:10000			Li-Cor	926_32212
anti-rabbit IRDye800CW anti-mouse	IgG	Donkey	1:10000			Li-Cor	926_32213
Anti-Mouse-IgG-HRP	IgG	Horse	1:1000			CST	7076

SI Table 5. qRT primer sequences used in this study. Related to STAR methods. All primers were purchased from Sigma except Cdkn2a, which was designed using Primer3.

Gene	Forward primer sequence (5'-3')	Reverse primer sequence (5'-3')	Source
Pdgfra	GAGATTATGAATGTGCTGCC	TTTCTCGTGAACAGAAATGC	Sigma
Ascl1	AAACAAGGGAAGAGGAAAAG	CATTGAATCTAAGTCCTGGTG	Sigma
Sox6	TCCCAATTTTTCCACATGAC	GTTATCACCTGGCTTGTATG	Sigma
Enpp6	AATTTGTCTCTCCTTTGACC	CTTTCTGGACATCAGATAGC	Sigma
Cnp1	TTTCAAGAAAGAGCTTCGAC	TAAGATCTCCTCACCACATC	Sigma
Cdkn2a	TCGTGCGGTATTTGCGGTAT	TAGTCTCGCGTTGCCAGAAG	This study
Tbp	CATCATGAGAATAAGAGAGCC	GGATTGTTCTTCACTTTGG	Sigma
Prkaa2	ccaaattatgcagcaccggag	acgtgctcatcgtcgaacg	This study
